# Biarticular muscles are most responsive to upper-body pitch perturbations in human standing

**DOI:** 10.1038/s41598-019-50995-3

**Published:** 2019-10-10

**Authors:** Christian Schumacher, Andrew Berry, Daniel Lemus, Christian Rode, André Seyfarth, Heike Vallery

**Affiliations:** 1 Lauflabor Locomotion Laboratory, Institute of Sport Science, Centre for Cognitive Science, Technische Universität Darmstadt, Darmstadt, 64289 Germany; 2 Delft Biorobotics Lab, BioMechanical Engineering, Delft University of Technology, Delft, 2628 CD The Netherlands; 3 Friedrich-Schiller-Universität Jena, Institute of Zoology and Evolutionary Research, Jena, 07743 Germany

**Keywords:** Physiology, Biomedical engineering, Motor control

## Abstract

Balancing the upper body is pivotal for upright and efficient gait. While models have identified potentially useful characteristics of biarticular thigh muscles for postural control of the upper body, experimental evidence for their specific role is lacking. Based on theoretical findings, we hypothesised that biarticular muscle activity would increase strongly in response to upper-body perturbations. To test this hypothesis, we used a novel Angular Momentum Perturbator (AMP) that, in contrast to existing methods, perturbs the upper-body posture with only minimal effect on Centre of Mass (CoM) excursions. The impulse-like AMP torques applied to the trunk of subjects resulted in upper-body pitch deflections of up to 17° with only small CoM excursions below 2 cm. Biarticular thigh muscles (biceps femoris long head and rectus femoris) showed the strongest increase in muscular activity (mid- and long-latency reflexes, starting 100 ms after perturbation onset) of all eight measured leg muscles which highlights the importance of biarticular muscles for restoring upper-body balance. These insights could be used for improving technological aids like rehabilitation or assistive devices, and the effectiveness of physical training for fall prevention e.g. for elderly people.

## Introduction

Dealing with typical perturbations (e.g. pushing, stumbling or walking on uneven ground) comprises coordinating multiple joints^1^. For this, biarticular muscles (that span two joints) might play a key role. In contrast to monoarticular muscles (spanning one joint), biarticular muscles contribute strongly to the leg force that acts perpendicular to the leg axis^2^. This seems to make biarticular muscles especially suitable for postural control because the perpendicular component of leg force regulates the angular momentum.

In accordance with this, in static experiments humans mainly used biarticular thigh muscles to control the direction of the Ground Reaction Force (GRF)^3–6^. This ability might also be exploited to achieve stable walking by directing the GRF towards a point above the Center of Mass (CoM)^7–10^. Further, humans responded quickly with hamstring activity to a perturbation of angular momentum in a stumbling experiment^11^. Simulations and robotic demonstrators revealed the potential of biarticular structures (e.g. springs or muscles) to stabilise the trunk during walking and generate appropriate leg swing motions^12–15^. However, experimental evidence for the actual use of biarticular muscles for upper-body balance in humans is still missing. Based on their ability to generate appropriate combinations of required hip and knee torques^4–6,16^, we hypothesised that biarticular thigh muscles would react strongly to a perturbation of upper body angular momentum during quiet standing.

**Figure 1:**
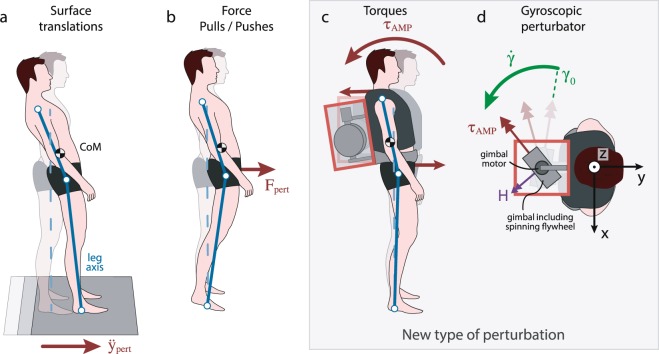
Exemplary perturbation types: Schematic visualisation of Center of Mass (CoM), leg, and upper-body kinematics (transparent: before perturbation, solid: after perturbation). **(a)** Back and forth surface translations result in a whole-body balance perturbation by producing first an acceleration followed by a deceleration. **(b)** Force pushes or pulls can result in irregular body postures with small upper body perturbations. **(c)** Torque perturbation: the generated torque on the upper body (curved red arrow) is equivalent to a force pair (straight red arrows) with zero net horizontal force. This type of perturbation results mainly in rotational acceleration of the upper body. Minimal CoM excursions occur due to the muscular coupling of the upper body to the leg. **(d)** The Angular Momentum Perturbator (AMP) creates external torques by rotating a spinning flywheel (angular momentum *H*, purple arrow) around a perpendicular gimbal axis (here: longitudinal, angular velocity $\dot{{\boldsymbol{\gamma }}}$, green arrow, initial gimbal position *γ*_0_). The created torque *τ*_AMP_ is exerted in the direction perpendicular to both, the rotation of the spinning flywheel and the gimbal axis, and rotates together with the gimbal (red arrow).

Perturbing a system and investigating its response is a standard method to analyse the system’s dynamics^17^. To study human balance strategies, unexpected and specific mechanical perturbations such as surface translations (Fig. 1a) have frequently been used to study the human balance response to slipping^17–20^. Recently, research groups have also applied pulls and pushes at the hip or at the shoulder^21–27^ to resemble other common perturbation scenarios (Fig. 1b). By generating a horizontal force, these systems perturb balance of the body as a whole^28^. Pushes/pulls and surface movement perturbations require corrections of angular momentum, i.e. keeping the CoM within the base of support and restoring the upright upper-body orientation. Thus, such perturbations necessitate a complex response of the neuro-musculoskeletal system, which might involve multiple response mechanisms. This might complicate or hamper the interpretation of the role of biarticular muscles for upper-body balance.

For the first time, we propose to exert a pure torque, or force couple, on the upper body. Applying a pure torque without a translational (horizontal force) component entails that the whole-body CoM position is only minimally influenced and reduces artefacts of cross-talk between different balance strategies. For this purpose, we use an Angular Momentum Perturbator (AMP)^29^. This system opens a new category in the framework of Shirota *et al*.^30^ and enables a specifically targeted perturbation (Fig. 1c), making it a suitable tool to study upper-body balance response strategies while maintaining a natural leg axis alignment. The system is portable – the subject can move freely without being bound to a treadmill or a frame construction – and is capable of generating powerful torque bursts.

In this study, we contribute to the ongoing research of human motor control during non-stepping balance recovery by examining the muscular response of major leg muscles for two major perturbation directions: (1) positive torque perturbations resulting in a forward upper-body pitch and (2) negative torque perturbations resulting in a backward pitch. Compared to other commonly used perturbation types, the AMP produced perturbations with a distinct upper-body pitch and only a small shift in CoM position, allowing us to assess the contribution of different leg muscles (using electromyographic data (EMG)) for restoring the upper-body balance in near-absence of whole-body balance corrections.

## Methods

### Angular Momentum Perturbator (AMP)

The AMP is worn like a backpack and contains a *control moment gyroscope*, an actuator that exerts torques by manipulating the angular momentum of an internal flywheel. This flywheel is mounted to a motorised gimbal frame (Fig. 1d), which enables the flywheel to be reoriented with respect to the wearer.

For the present study, we modified the AMP prototype described previously^29^ to reduce its mass. After replacing the gimbal motor and transmission, the system weighed 16 kg and was capable of exerting a maximum torque of 53 Nm.

The AMP exerts torques on the wearer (***τ***_AMP_) by changing the direction or magnitude of the angular momentum of the flywheel (***H***), described in detail by e.g. Schaub *et al*.^31^. Changing the direction by rotating the gimbal or rotating the trunk produces *gyroscopic* torques proportional to the angular velocities of the gimbal ($\dot{{\boldsymbol{\gamma }}}$) and trunk of the wearer (***ω*** = [*ω*_*x*_, *ω*_*y*_, *ω*_*z*_], using the frame definitions of Fig. 1d). When exploiting this gyroscopic effect, the gimbal motor applies a torque (***τ***_*g*_) in the gimbal axis to modulate $\dot{{\boldsymbol{\gamma }}}$; however, in doing so, this changes the magnitude of ***H*** and is experienced by the wearer as an opposite reaction torque in the same axis. The total torque is thus the sum of these components:

1 $${{\boldsymbol{\tau }}}_{{\rm{A}}{\rm{M}}{\rm{P}}}(t)=-\,\dot{{\boldsymbol{H}}}\,(t)=\mathop{\underbrace{-\,(\dot{{\boldsymbol{\gamma }}}(t)+{\boldsymbol{\omega }}(t))\times {\boldsymbol{H}}(t)}}\limits_{{\rm{g}}{\rm{y}}{\rm{r}}{\rm{o}}{\rm{s}}{\rm{c}}{\rm{o}}{\rm{p}}{\rm{i}}{\rm{c}}\,{\rm{e}}{\rm{f}}{\rm{f}}{\rm{e}}{\rm{c}}{\rm{t}}}\,-\mathop{\underbrace{{{\boldsymbol{\tau }}}_{g}(t)}}\limits_{{\rm{g}}{\rm{i}}{\rm{m}}{\rm{b}}{\rm{a}}{\rm{l}}\,{\rm{m}}{\rm{o}}{\rm{t}}{\rm{o}}{\rm{r}}}.$$

By design, the gyroscopic torque is generally much larger than the gimbal torques (***τ***_*g*_) to allow the use of a relatively small gimbal motor and reduce power requirements. Since this gyroscopic torque depends not only on the controlled gimbal velocity ($\dot{{\boldsymbol{\gamma }}}$), but also on the angular velocity of the wearer ***ω***, it is in general only partially controllable. However, in the present study, since ***ω*** is relatively small (especially during quiet standing), the uncontrolled gyroscopic torques were typically at least one order of magnitude smaller than those due to $\dot{{\boldsymbol{\gamma }}}$ and were mostly considered negligible.

When generating controlled gyroscopic torques by rotating the gimbal, the direction of ***τ***_AMP_ simultaneously changes with the gimbal angle (***γ***). In a human-fixed frame (unit vectors $({\hat{{\bf{e}}}}_{x},{\hat{{\bf{e}}}}_{y},{\hat{{\bf{e}}}}_{z})$ attached to frame (***x***, ***y***, ***z***), Fig. 1d), the total perturbation torque ***τ***_AMP_ consists of components in all three directions:

2 $$\begin{array}{ccc}{{\boldsymbol{\tau }}}_{{\rm{A}}{\rm{M}}{\rm{P}}}(t) & = & -\mathop{\underbrace{{\tau }_{t}(t)\,\cos \,(\gamma (t)){\hat{{\boldsymbol{e}}}}_{x}}}\limits_{{\rm{p}}{\rm{i}}{\rm{t}}{\rm{c}}{\rm{h}}\,{\rm{c}}{\rm{o}}{\rm{m}}{\rm{p}}{\rm{o}}{\rm{n}}{\rm{e}}{\rm{n}}{\rm{t}}}-\mathop{\underbrace{{\tau }_{t}(t)\,\sin \,(\gamma (t)){\hat{{\boldsymbol{e}}}}_{y}}}\limits_{{\rm{r}}{\rm{o}}{\rm{l}}{\rm{l}}\,{\rm{c}}{\rm{o}}{\rm{m}}{\rm{p}}{\rm{o}}{\rm{n}}{\rm{e}}{\rm{n}}{\rm{t}}}+\mathop{\underbrace{({\omega }_{t}(t)H(t)-{\tau }_{g}(t)){\hat{{\boldsymbol{e}}}}_{z}}}\limits_{{\rm{y}}{\rm{a}}{\rm{w}}\,{\rm{c}}{\rm{o}}{\rm{m}}{\rm{p}}{\rm{o}}{\rm{n}}{\rm{e}}{\rm{n}}{\rm{t}}}\\  & \approx  & -\dot{\gamma }(t)\,H\cos \,(\gamma (t)){\hat{{\boldsymbol{e}}}}_{x}-\dot{\gamma }(t)\,H\sin \,(\gamma (t)){\hat{{\boldsymbol{e}}}}_{y}+({\omega }_{x}(t)\,H\cos \,(\gamma (t))-{\tau }_{g}(t)){\hat{{\boldsymbol{e}}}}_{z},\end{array}$$

where *τ*_*t*_(*t*) = ($\dot{{\boldsymbol{\gamma }}}$(*t*) +* ω*_*z*_(*t*))*H*(*t*) ≈ $\dot{{\boldsymbol{\gamma }}}$(*t*)*H*(*t*) and *ω*_*t*_(*t*) = *ω*_*x*_(*t*)cos(*γ*(*t*)) + *ω*_*y*_(*t*)sin(*γ*(*t*)) *≈* *ω*_*x*_(*t*)cos(*γ*(*t*)). All non-bold variables indicate the signed scalar magnitudes of the vector quantities defined previously, and the magnitude of the flywheel angular momentum was approximately constant throughout all experiments (*H*(*t*) ≈ *H*).

To generate a torque of a specific magnitude and direction requires inversion of Eq. (2) to produce a reference gimbal motion, described in general by Berry *et al*.^32^. For discrete open-loop perturbations, this inversion is simplified, and, for a given initial gimbal angle (*γ*_0_) and desired torque profile, the final angle (*γ*_*f*_) can be computed in advance. To produce a torque primarily in the sagittal plane (around the *x*-axis) and limit the component in the frontal plane (around the *y*-axis), the range of gimbal rotation was constrained and *γ*_0_ was chosen such that *γ* ≈ 0 when the magnitude of ***τ***_AMP_ was maximal (Fig. 1d).

**Figure 2:**
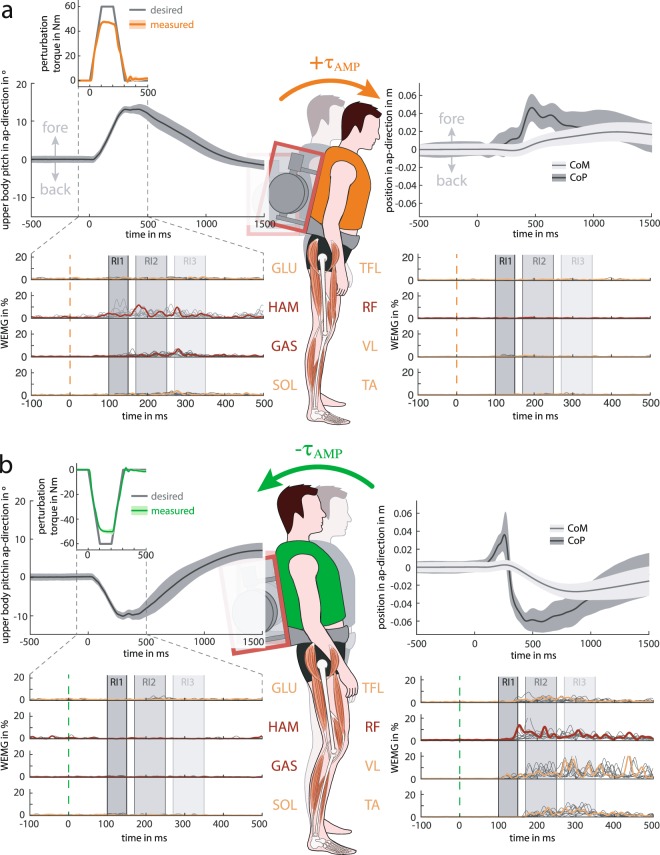
AMP generated (**a**) positive and (**b**) negative torque perturbations resulting in forward and backward pitch of the upper body, respectively. Change in upper-body pitch angle of one subject (mean and standard deviation from the last 5 trials × 2 gimbal configurations) with respect to the initial upper-body posture (top left). The inset shows the desired (grey, 100 rise, hold and fall time) and actual pitch perturbation torque profile (orange/green: mean and SD). Change in sagittal CoM (grey: mean and SD) and CoP position (black: mean and SD) of the same subject with respect to the initial positions (top right). Exemplary EMG of the same subject (bottom panel): filtered and normalised signals of all trials (grey) and one individual response of mono- (yellow) and biarticular muscles (red).

The perturbation torque ***τ***_AMP_ was selected to be a symmetric trapezoidal profile (Fig. 2 inset), consisting of a peak torque of 60 Nm with a rise time, hold time, and fall time each of 100 ms. This shape reflects the finite ability of the gimbal motor to accelerate or decelerate rotation of the gimbal structure to produce the gyroscopic effect. A gimbal motor torque (*τ*_*g*_) of approximately 12 Nm was necessary to generate the desired perturbations. Because the roll (frontal) torque components alternated sign throughout the perturbation, all perturbations were repeated with the gimbal inverted (rotated by 180°) to also generate the opposite pattern (of roll torque component) with similar pitch torque component for comparison.

### Experimental protocol

The experimental protocol consisted of three successive sets of measurements involving different settings. In the first setting, the subjects were asked to stand still for 60 seconds with arms crossed over the chest and without wearing the perturbator (‘Unloaded Standing’). In the second setting, they repeated the same task but wore a safety harness and the AMP with spinning flywheel, but no active torque perturbations (‘Loaded Standing’). In the final setting of standing, a series of 48 trials were executed involving 40 trials of active torque perturbation of the AMP (‘Perturbation Trials’) and 8 trials which lacked a perturbation (‘Control Trials’). The 40 Perturbation Trials consisted of 4 conditions with 10 repetitions each: positive and negative torque directions with both original (*γ*_0_ = 0°) and inverted (*γ*_0_ = 180°) initial gimbal orientations. Conditions of positive and negative torque direction (inducing forward and backward upper-body pitch, respectively) were chosen to investigate a potential direction dependency. Since the AMP generates a complex perturbation that acts in multiple planes (mainly sagittal but also in the frontal plane), the initial gimbal position was altered to investigate potential side effects of the roll torque component. Due to relatively long gimbal re-positioning times before every trial (to reach the initial gimbal position), a blocked protocol was preferred over a fully randomised protocol (‘Block 1’ with original and ‘Block 2’ with inverted gimbal position). This was done to reduce the measurement time and subject fatigue. Within the two blocks, both the direction and timing of the active perturbations were randomised. Subject were given time to rest in between measurements. For referencing, subjects conducted ten additional walking trials (distance approx. 7 m) at preferred walking speeds (1.20 ± 0.14 ms^−1^, Mean ± SD) without any additional loads.

To prevent fall-related injury during the perturbations, a safety harness was used at all times when the AMP was worn. The harness was attached to the Rysen body weight support system (Motekforce Link, Amsterdam, The Netherlands), which is capable of actively detecting and arresting falling motions of the subject^33^. To avoid vertical unloading forces during the measurements, the Rysen system was lowered and locked in a position where the harness straps were slack during both quiet and perturbed standing, but could still prevent falls if balance could not be successfully recovered.

### Data collection and processing

#### Subjects

Seventeen healthy adult subjects (two female) participated in the study. All subjects volunteered to participate in the study in summer 2018 and gave written informed consent in advance of the experiment. The experimental protocol was approved by and performed in accordance with the relevant guidelines and regulations of the Human Research Ethics Committee of the Delft University of Technology (Project ID: 350). Before the experiment, all participants gave their consent and filled in the revised Waterloo Footedness Questionnaire^34^, which was used to assess limb dominance regarding stabilisation tasks.

We collected EMG, kinetic, and kinematic data of the subject. All measurement devices including the data logging of the AMP were synchronised by a manual trigger signal. Before processing the data, one female subject was excluded from the data analysis due to repetitive stepping responses during the experiments. A further five (male) subjects were excluded due to missing EMG data or missing marker data that impeded a calculation of the CoM. Only the data of the remaining eleven subjects (one female), of age 34 ± 14 years (Mean ± SD), weight 74.8 ± 12.5 kg, and height 1.81 ± 0.08 m, were considered for further analysis.

#### Measured data

Sixteen surface EMG electrodes (Trigno, Delsys Inc., Natick, USA) were used to record at 2000 Hz the electrical activity of relevant leg muscles. The set of electrodes were placed on the following muscles of each leg: tibialis anterior (TA), soleus (SOL), gastrocnemius lateralis (GAS), vastus lateralis (VL), rectus femoris (RF), biceps femoris long head (HAM), tensor fasciae latae (TFL) and gluteus maximus (GLU) (see Supplementary Fig. S1). To ensure a good electrical connectivity, the skin was prepared following the SENIAM recommendations^35^. After attachment, electrode locations were checked for voluntary muscle signals and low noise values. The raw EMG was band-pass-filtered with cut-off frequencies of 20 Hz (high-pass) and 450 Hz (low-pass), further rectified and low-pass filtered at 50 Hz. For each muscle and subject, the filtered EMG signals were normalised by the mean filtered background activity recorded during unloaded level walking (WEMG), and expressed as a percentage of WEMG.

Individual GRF of both legs were measured at a frequency of 1000 Hz (3^rd^-order analogue Butterworth low-pass filter with 500 Hz cut-off frequency) using two force plates (9260AA3, Kistler Holding AG, Winterthur, Switzerland), each for a single leg. These were combined to compute the Whole-body Center of Pressure (CoP).

An inertial measurement unit (IMU) (MPU-9250, InvenSense, San Jose, USA) within the AMP and a motion capture system (Qualisys, Gothenburg, Sweden) were used to collect kinematic data (200 Hz) of the subject and the AMP. Nineteen reflective markers were placed at relevant body locations (see Supplementary Fig. S1): the tragion (TRA), 7th cervial vertebrae (C7), acromion (ACR), greater trochanter (GTR), lateral femoral condyle (LFC), fibulare (FIB), lateral melleoius (LM), calcaneus (CAL), 1st metatarsal head (MT1), and 5th metatarsal head (MT5). The CoM of the AMP was estimated by suspending the AMP from a single attachment point, and measuring the point of intersection of the vertical axis as the attachment point was changed. Four addition markers were placed on the rigid and static frame of the AMP such that their mean position resembled the CoM of the AMP. All kinematic data was upsampled to 1000 Hz by linear interpolation and filtered using a zero-lag 4^th^-order low-pass filter with a cut-off frequency of 6 Hz.

#### Computing outcome measures

Since the unintended roll torque components created only slight opposing asymmetric behaviours with similar magnitude in both conditions of initial gimbal positions (‘Block 1’ vs. ‘Block 2’, see Supplementary Figs S9–S14), both sets were merged for comprehensibility reasons. Outcome measures, such as mean and SD of kinematic and kinetic data, were calculated from both blocks together.

Mean muscle stimulation of ‘Unloaded Standing’ and ‘Loaded Standing’ was calculated for a 20 s period of quiet standing. The mean signal of the preceding 500 ms prior to perturbation onset (quiet standing) was used as ‘Pre-Perturbation’ activation. From this signal, the mean of each muscle and subject of the last 10 trials per block (‘Block 1’ and ‘Block 2’) was compared against the condition of ‘Loaded Standing’ to evaluate muscle activity changes due to anticipation.

In order to account for temporal variability of the muscular activity for all ‘Perturbation Trials’, three time intervals were chosen to evaluate the appearance of medium-latency response (RI1: from 100 ms to 150 ms) and long-latency responses (RI2: from 170 ms to 250 ms, RI3: from 270 ms to 350 ms) that involve contributions from supra-spinal centres or poly-synaptic reflex responses^36–38^. The relative reflex response of each muscle was defined as the difference between the mean EMG in the response intervals and corresponding mean EMG in the ‘Pre-Perturbation’ interval of the same trial, each expressed as a percentage of WEMG. As adaptation processes of EMG responses have been found to settle after about 5 trials^39^, we considered only the last 5 of the 10 trials per condition, in order to reduce the effects of adaptation processes. We used the Grubbs’ test (with a significance level of *α* = 0.05) to identify and remove unphysiological EMG values (outliers), stemming from e.g. physical collisions of the hip belt and hip muscle electrodes. To evaluate the muscular reflexes, we first computed the mean relative reflex response of all analysed trials per subjects (last 5 of the 10 trials per condition, combining ‘Block 1’ and ‘Block 2’) before performing the statistical analysis. Outcome measures of pooled data are reported by grand means and SD of the averaged subject data. Data analysis was done using Matlab 2016b (The MathWorks Inc., Natick, USA).

### Statistical analysis

We compared different joint angles and activity levels between ‘Unloaded Standing’ and ‘Loaded Standing’ as well as ‘Loaded Standing’ and ‘Pre-Perturbation’ conditions. First we evaluated the normality of the residuals by the Shapiro-Wilk test. If they were normally distributed we applied a paired two-sided t-test. In other cases we tested for differences with the non-parametric two-sided Wilcoxon signed-rank test. All comparisons were performed using a 5% significance level.

For comparisons of the relative reflex response, a repeated measures analysis of variance (rmANOVA) with between (‘Direction’, ‘Side’ and ‘Muscles’) and within subject factors (‘Response Intervals’) was performed. Shapiro-Wilk tests confirmed normality of the data. If Mauchly’s test for sphericity revealed that homogeneity of the data was not given, the Greenhouse-Geisser correction was used. Comparisons were performed using a 5% significance level. If the rmANOVA showed significant interaction effects, post-hoc tests were computed with the Tukey’s Honest Significant Difference procedure to adjust for multiple comparisons.

## Results

We perturbed upper-body posture during quiet standing by applying external torques – instead of forces – created by a new type of perturbation device, the AMP. This was done to specifically investigate the role of biarticular leg muscles used to control upper-body balance.

The outcome measures of both conditions of initial gimbal positions (‘Block 1’ and ‘Block 2’) were merged before computing statistics. The results will thus be presented for both main perturbation directions: positive torque perturbation (*τ*_AMP_ > 0) with forward upper-body pitch and negative torque perturbation (*τ*_AMP_ < 0) with backward upper-body pitch. In the following, we will focus on the results of the left leg (the dominant leg for stabilisation of all subjects) as leg behaviour (see Supplementary Figs S12–S14 and Table S2) and response characteristics (‘Side’ main effect of rmANOVA: *F*(1,314) = 0.18, *p* = 0.676) were similar for both sides.

### AMP produced dynamic and reproducible torque perturbations

To verify that the AMP generated appropriate perturbation profiles, the calculated gyroscopic torque (based on measured gimbal angular velocity $\dot{{\boldsymbol{\gamma }}}$) was compared to the desired profile (Fig. 2 insets). For both perturbation directions, the AMP was able to generate repeatable perturbations that closely resembled the shape of the desired profile. The rise and fall dynamics were tracked accurately, but the realised peak torque (53 Nm) fell lower than the target of 60 Nm. For both perturbation directions, the small standard deviation (SD) of the measured profile confirms a consistent perturbation generation over multiple trials (Fig. 2 insets).

### Torque perturbations resulted in specific upper-body pitch perturbations

In order to study the resulting posture of the subjects, we evaluated upper-body pitch (sagittal-plane rotation), whole-body CoM and CoP (top panels in Fig. 2a). Following the positive torque perturbation, the upper-body segment pitched forward with a peak rotation of 15° to 17° with respect to the initial posture before perturbation onset. The peak pitch was reached after 300 ms to 400 ms after perturbation onset, and about 1000 ms to 1500 ms were needed to recover the initial posture. This response was accompanied by a backwards movement of the hip joint (see Supplementary Fig. S4). In contrast to the substantial upper-body rotation, the CoM moved forward by only about 1 cm to 2 cm. The CoM progression started after 400 ms. The mean CoP moved anterior by 4 cm to reach its peak after 500 ms. The subjects’ mechanical response within the first 1000 ms consisted of a distinct early upper-body pitch, mainly due to hip flexion within first 500 ms (see Supplementary Fig. S2), followed by a delayed, but subtle CoM forward sway.

For negative torque perturbations, the resultant backwards pitch of the upper body (top panels in Fig. 2b) was smaller (approx. −12°) compared to the forward perturbations and appeared at similar timing (300 ms to 400 ms after perturbation onset). During upper-body pitch, the hip joint moved forward (see Supplementary Fig. S4). Again delayed, the CoM moved posterior to a peak of about −2 cm from the initial position. A distinct CoP pattern was measured: for the time of backward acceleration of the upper body, the CoP moved forward before quickly moving backwards to around −5 cm. In the backwards condition, the upper-body rotation was not achieved through hip extension (remained in initial posture), but by knee bending such that upper body and thigh segments pivoted together around the knee joint (see Supplementary Fig. S2).

### Influence of AMP weight, noise, and vibrations

**Figure 3:**
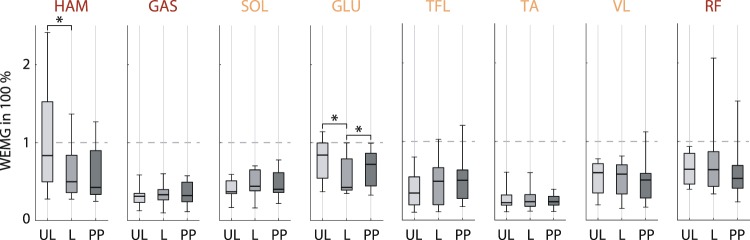
Influence of AMP artefacts and prestimulation activity. Boxplots of muscular activity of the left leg in ‘Unloaded Standing’ (UL), ‘Loaded Standing’ (L) and the mean ‘Pre-Perturbation’ activation of the last 10 trials per block (‘Block 1’ and ‘Block 2’) for all 11 subjects. Reported EMGs (WEMG) are the mean intervals of the filtered EMG signals normalised by the muscle’s mean activity during walking (see Methods). Results are presented with significant (^*^p < 0.05) comparisons of the paired t-test or two-sided Wilcoxon signed-rank test.

Next, we tested the extent to which the weight, noise, and vibrations of the AMP influenced the initial posture and the EMG. Wearing the AMP led to a more bent hip (−6.2°, *t*(10) = −5.528, *p* < 0.001) and more extended knee (2.1°, *t*(10) = 3.652, *p* = 0.002) while no difference was found for the ankle joint (see Supplementary Table S1). By comparing EMG data of the two conditions of quiet standing ‘Unloaded Standing’ and ‘Loaded Standing’ changes of muscle stimulation were identified (Fig. 3). The additional weight, noise, and vibration of the rotating flywheel of the AMP resulted in significant reductions of muscular activity in GLU (unloaded: 78 ± 28% WEMG, loaded: 57 ± 24% WEMG, *Z* = −2.934, *p* = 0.003) and HAM (unloaded: 108 ± 75% WEMG, loaded: 62 ± 34% WEMG, *Z* = −2.490, *p* = 0.013) of the left leg – reflecting the reduced demand of muscle generated hip extension torque to maintain similar upper-body postures – as well as VL of the right leg (see Supplementary Table S2). No other significant differences were found.

### Prestimulation of monoarticular hip muscles to prepare for perturbations

We compared the muscular activity in the ‘Loaded Standing’ and the ‘Pre-Perturbation’ activation to assess if subjects increased muscular activity in anticipation of perturbation, despite the random timing and direction (Fig. 3). Significant changes of the monoarticular hip muscles GLU of the left leg (increased by: 8 ± 13% WEMG, *Z* = 2.134% WEMG, *p* = 0.033). From all other muscles, only TFL in the right leg showed a significant reduction (see Supplementary Table S2).

### Biarticular thigh muscles were major contributors to recover upper-body balance

We next tested which muscles showed the highest response activity to identify the most reactive muscles. For both perturbations, early EMG responses within the first 100 ms after the perturbation were not found. In the analysed response intervals, both perturbation directions evoked different muscular responses (‘Direction’ main effect: *F*(1,314) = 173.8, *p* < 0.001).

**Figure 4:**
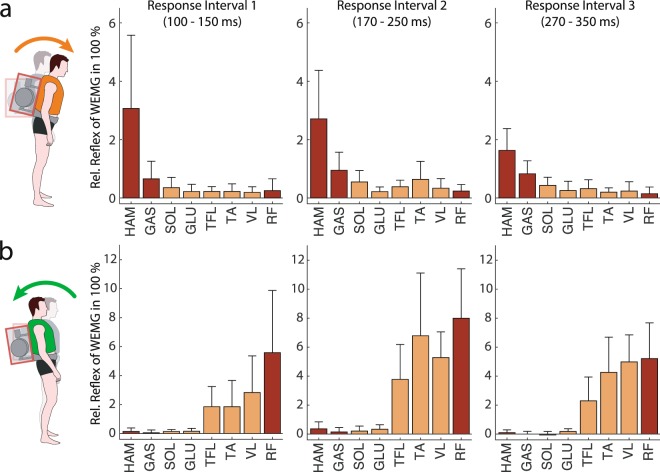
Reflex activity of mono- (yellow) and biarticular muscles (red) for (**a**) positive and (**b**) negative torque perturbations in different response intervals. Grand mean and SD of averaged relative reflex responses of the left leg (last 5 trials per condition × 2 gimbal configurations) for all 11 subjects. Relative reflex activity in each response interval is computed with respect to ‘Pre-Perturbation’ and normalised by the muscle’s mean activity during walking (see Methods).

For positive torque perturbation with forward upper-body pitch, we found the highest muscle activity levels for the biarticular thigh muscle HAM, the biarticular ankle muscle GAS and the monoarticular ankle muscle SOL. HAM response activity started already in the mid-latency response window (RI1) and lasted until RI3 with up to 366 ± 271% WEMG. GAS (up to 128 ± 61% WEMG) and SOL (up to 101 ± 35% WEMG) responded later in RI2 or RI3. By this, HAM was found to increase its activity (in RI1 and RI2) by more than double of its mean activity during walking (Fig. 4a) which was significantly higher than in all other muscles (see Supplementary Table S3).

In negative perturbations, the biarticular thigh muscle RF, the monoarticular ankle flexor TA and the monoarticular knee extensor VL were found to have the highest EMG amplitude with respect to their mean activity during normal walking. RF activity was high in all response intervals (up to 866 ± 341% WEMG). RF, TA (up to 706 ± 435% WEMG) and VL (up to 582 ± 178% WEMG) reached the highest response in RI2. TFL activity with a maximum of 428 ± 257% WEMG in RI2 increased by half of the RF increase. Since ‘Pre-Perturbation’ activity was similar for TFL, RF and VL, RF was also the muscle with the strongest and earliest increase in activity (Fig. 4b). Compared to all other muscles, RF reflex activity was significantly stronger than in all other muscles (see Supplementary Table S3). From a lower activity (24 ± 9% WEMG) before the perturbation onset, TA increased by 678 ± 433% WEMG in RI2, but only showed low increase in RI1.

Summarising these results, biarticular thigh muscles (HAM and RF) were found to have the highest activity in the earliest response interval (RI1), the highest overall amplitude with respect to their mean activity during walking and the highest increase compared to unperturbed standing (significantly higher than all other measured muscles). Ankle joint muscles like GAS, SOL or TA contributed moderately with delayed increase, mainly in RI2 and RI3. In contrast to biarticular thigh muscles, monoarticular hip muscles GLU and TFL increased only slightly or moderately in response to the evoked upper-body pitch deflection.

## Discussion

Previous research identified beneficial features of biarticular muscles that could potentially contribute to recover balance after postural perturbations^4,6,40,41^. Experimental evidence that highlights the specific contribution of biarticular muscles to upper-body balance is however missing. To fill this gap of knowledge, this study examined the muscular response of major leg muscles for recovering from artificial torque perturbations to the upper body. For the first time, an Angular Momentum Perturbator (AMP) was used to directly apply reproducible torques to the upper body during quiet standing without a translational (e.g. horizontal foreward/backward force) component of other typical perturbations like pushes or pulls. By analysing the mechanical response of the subjects and EMG of major leg muscles, we found that

1. Biarticular thigh muscles HAM and RF showed the strongest increase in activity in response to upper-body balance perturbations of all measured leg muscles;
2. AMP torques resulted in nearly isolated upper-body postural alignment perturbations.

### Biarticular muscles and upper-body balance

In our study, biarticular thigh muscles showed the strongest reflex responses. Perturbing the upper-body challenged the motor control system to re-erect the upper-body. The positive perturbation created forward upper-body pitch (about 17°). Restoring the original body posture requires hip extension torques. These would cause knee overextension which must be counteracted by knee flexion torques. The biarticular HAM muscle group delivers simultaneous hip extension and knee flexion torques. This might explain our finding that the HAM showed the strongest EMG increase of all measured muscles. The negative perturbation led to backwards upper-body pitch (about −12°). Conversely, to re-erect the upper-body, hip flexion and knee extension torques are required. The biarticular RF muscle group delivers simultaneous hip flexion and knee extension torques. Correspondingly, the strongest EMG response was found for the biarticular RF.

Our results confirmed our hypothesis of a strong involvement of biarticular thigh muscles for restoring upper-body balance. This result agrees well with the previously suggested torque-based (net hip minus knee torque; extension torques are positive) control scheme of biarticular muscles for joint coordination in tasks involving opposing changes in hip and knee torques. For example, high HAM and RF activity was found during GRF direction manipulations in isometric and isokinetic conditions^4,6^, load-lifting^42,43^, cycling^44^ as well as in the swing leg during walking and running^16^. In contrast to these studies involving planned movements, our experiment targeted perturbation recovery. Studies in this area involving surface translations and force pushes/pulls^17–27^ show that, among other muscles, biarticular muscles react to compensate for these perturbations. Our experiments refined these findings by providing evidence for a strong reactive involvement of biarticular thigh muscles to compensate torque perturbations applied at the upper body. This can most clearly be observed for the positive torque perturbation.

The positive and the negative perturbation generated different responses that might be related to joint range-of-motion constraints. While the positive perturbation lead to strong hip flexion (about 16°) and only small knee extension (about 2°, see Supplementary Fig. S2), the negative perturbation generated moderate hip extension (about 6°) and strong knee flexion (about 14°, see Supplementary Fig. S2). From a straight configuration as reference, the range of motion of the hip is approximately 120° flexion and only 10° hip extension^45^. The knee constraint is even more strict with approximately 140° flexion and 2° extension^45^. While our positive perturbation induced mainly upper body forward pitch, the negative perturbation resulted in a backwards pitch of the upper body and the thigh segments together. Thus, the asymmetric perturbation responses reflect the joint range-of-motion constraints.

The observed asymmetric perturbation responses induced different torque requirements. In the case of the positive perturbation, biarticular HAM activity seems to be sufficient to re-erect the upper body. For negative perturbations, in addition to pronounced RF activity we observed increases in activity of monoarticular knee extensors (VL) and to a lesser extent of the functionally monoarticular hip flexor muscles (TFL). It seems that despite its ability to provide for simultaneous knee extension and hip flexion torques, RF may not be able to meet the torque requirement at these joints. RF has approximately half of the physiological cross-sectional area and thus half the force capacity of HAM^46^. In addition, it has a lower lever arm at the hip than HAM^47,48^, which may explain the increase in activity of the functionally monoarticular hip flexor TFL. The different capacity of the biarticular HAM and RF muscles to generate joint torques may stem from an adaptation towards their daily use, reflecting a dominating use of HAM in walking and running due to a forward lean of the upper body^49^. For less intense negative torque perturbations, we expect to see disproportionally less pronounced knee flexion (reducing the kinematic difference between positive and negative perturbations) and reduced activity of monoarticular muscles. RF torques might then be sufficient to re-erect the upper body.

During perturbations in both directions, different mechanisms led to opposing CoM and CoP patterns. In positive perturbations, the upper body pitched forward about the hip. During this early response, extension hip torques were generated by increased HAM activity. The hip coupling to the leg (and subsequently to the ground) resulted in a delayed acceleration of the CoM in anterior direction (after 400 ms). The CoP travel is due to ankle extension torques provided by the SOL to counteract the forward movement of the CoM. During the backward perturbation, the upper body rotated backwards by extensive bending at the knee joint. This induced a pronounced eccentric stretch of the pre-activated SOL resulting in an instantaneous increase in muscle force^50^. This explains the initial anterior CoP travel (within the first 300 ms to 400 ms). This, however, is a critical condition for balance, because both the required hip flexion (to realign the upper body) and the ankle extension accelerate the CoM backwards^51^. To counter this, immediate deactivation of the SOL and activation of the TA would be required which we also observed in the EMG responses (RI3 in Fig. 4b). Further proof of these interpretations requires modelling and simulation of the neuro-muscle-skeletal system.

Reported EMG signals might contain cross-talk from other muscle groups. For example, the RF might have been contaminated by activity from the vastus intermedius^52^. Using needle electrodes might be a more reliable alternative. However, due to their invasive nature, the intramuscular electrodes require technical expertise, usually more time for preparing the subject and can create discomfort^53,54^, which in turn might influence the behaviour of the subject. By following the SENIAM recommendations^35^ for skin preparation and electrode placement, and by utilising appropriate surface electrodes^55,56^, we aimed at reducing the influence of cross-talk to a minimum.

Our experiments show that the biarticular thigh muscles strongly relate to postural upper-body control in standing. We speculate that these muscles could also play a key role for upper-body balance in walking. During walking, GRFs intersect in a point above the CoM which helps to stabilise the angular momentum of the whole body similar to a physical pendulum^7,9^. However, upper body stability is not achieved in this way, because the resulting forces acting on the upper body intersect below the CoM of the upper body^10^. Here, biarticular muscles can help to regulate the angular momentum of the upper body by applying torques via the stance leg^2,12,42,57–60^ or the swing leg^14–16^. The stabilising function of biarticular structures for the upper body during walking was demonstrated in a dynamically walking robot^13^. Further perturbation experiments will target the role of biarticular muscles for upper-body balance in walking.

### Anticipatory and reactive control strategies

When mechanical perturbations are applied, humans use different strategies to prepare or counteract the effect of the perturbation^17,61,62^. For instance, when a perturbation is expected or predicted, activity of antagonistic muscles can be increased simultaneously (co-contraction) in order to build up joint impedance before the perturbation onset^63^. This zero-delay mechanism is governed by feed-forward commands of supra-spinal centres and usually called ‘prestimulation’^50,64^. Especially when knowledge about the perturbation (e.g. intensity, timing or direction) is rare, joint impedance can help to preserve the body posture^65^.

In our experiment, an anticipatory co-contraction was not found. Only the monoarticular hip extensor GLU of the left leg showed an increased activity level in anticipation of the perturbation. In the right leg, the hip flexor TFL reduced its prestimulation. Explanations of this could be the influence of feed-forward commands^50^, e.g. limb dominance, or an unbalanced loading of both legs caused by the natural (lateral) sway^51^ that was also present in our study (see Supplementary Fig. S7). It is also likely that subjects adapted their prestimulation throughout the progress of the experiment. However, the results of Welch and Ting^66^, which found only modest changes of prestimulation in response to surface translations and only one to two trials with converging behaviour towards a preferred prestimulation activity, suggest that the influence of training-induced adaptation or habituation on prestimulation activity should be small.

Reactive responses (‘reflexes’) can (with a certain delay) respond to the perturbation by processing sensory feedback with only minimal or no prior knowledge^17,67,68^. We found that the temporal organisation of observed muscular responses remained (with only some exceptions) similar throughout the three response intervals. However, muscular responses were only found after a delay of 100 ms after the perturbation onset. Similar delays were found for surface translations^19,20^, while other (more sudden) upper-body perturbations resulted in shorter delays of e.g. 30 ms to 80 ms^69,70^. Since we measured an initial change of upper-body pitch 50 ms after onset, RI1 responses can be considered medium-latency while RI2 and RI3 denote long-latency responses^37^. Previous studies^71,72^ suggest that short-latency responses (not observed in our experiment) are induced by joint errors (e.g. stretch reflexes) and long-latency responses are the result of a task-level feedback. While these long-latency responses have been found to undergo modulations of task- and context-specific constraints^36,67,73–78^, they also reflect the inter-segmental coupling of joints^79–82^. For instance, perturbations to one joint can evoke responses in muscles that only act on unperturbed joints^18,82^. Such patterns were associated with pre-defined coordination patterns (e.g. muscle synergies) that are triggered by a certain stimulus and generate purposeful responses in the context of spartio-temporal or task-specific constraints^71,73,83^ also reflecting the requirements of everyday activities, such as controlling inertial effects of e.g. accelerated, neighbouring segments^79,82^. While reactive and anticipatory control strategies are usually combined^19,66,84–86^, our results show that biarticular muscles mainly follow a reactive control strategy.

Such long-latency responses can also undergo adaptation processes^71^. While the additional loading (the weight of the AMP) resembled a task that all subjects faced previously (e.g. when wearing a heavy backpack), pure torque perturbations are rare during activities of daily living. It is thus unlikely that subjects were familiar with the type of perturbation, resulting in adaptation or habituation effects from updating an internal model of the AMP and the applied torques. Prior information about the perturbation, learning, or training has been found to guide the selection of long-latency responses towards a strategy that also reduces muscular activity^19,39,66,87^. Also, previous studies found a wide variety of adaptation processes, ranging from approximately 5 trials^39^ to more than 50 trials^87^, so it is difficult to conclude that adaptation had ceased during the 10 trials per condition in our experiment. However, to reduce influences of learning on the reflex responses^66^, we considered only the last 5 of the 10 trials per condition. Still, we cannot exclude further training or learning of subjects. Nevertheless, given also other factors, such as an accelerated rate of fatigue during load carriage, the number of discarded trials must be kept small for practicality.

### Gyroscopic perturbation generation

The AMP is an example of ‘reactionless actuation’, in which torques are exerted by exchanging angular momentum with the actuator itself, rather than by exerting forces against the ground or an inertially-fixed object (e.g. an immobile robot transmitting forces via linkages or cables). This principle has both theoretical and practical benefits for perturbation experiments.

Specificity of a perturbation is difficult to achieve. Force-controlled systems are generally preferred over position-controlled systems due to their absence of kinematic constraints and more natural responses^88,89^. While it is, in principle, possible to generate a pure torque with a pair of opposing forces, it is, in practice, difficult to synchronise these forces and track the location of the CoM in real-time. Reactionless actuation is attractive because both synchronisation and alignment of the equivalent force couple happens inherently.

A practical benefit of reactionless actuation is that, since there is no necessity to exert forces against an inertially-fixed environment, the actuator can be entirely self-contained and even take the form of a wearable backpack, as in the AMP. This enables the AMP to be portable and suitable for overground experiments, and to be combined with other systems (e.g. treadmills, other perturbators) and measurement apparatus (e.g. motion capture, force plates) with minimal interference. To study other balance mechanisms in the future, wearable reactionless actuators might be placed on other parts of the body to perturb specific limbs. For various other (assistive) applications, such actuators have already been described for placement on the arms^90,91^, legs^92,93^, and distributed across the body^94^.

The gyroscopic torque vector rotates along with the gimbal creating – together with the desired pitch perturbation – roll torque components in the frontal plane (Eq. (2)). While the gimbal initial angle and total rotation were chosen to reduce the magnitude of this roll component, it was nevertheless present. In additional conditions in which the gimbal was rotated by 180°, the same pitch perturbations were produced but with the opposite roll components. From this, it was established that a lateral trunk lean did occur (up to 8°, see Supplementary Fig. S9), and that the load shifted from one leg to the other, but that this did not appear with asymmetric magnitudes or dependent of the side of dominance (see Supplementary Figs S10 and S11).

The frontal balancing of the upper body is mainly achieved by an (un-)loading mechanism of hip abductors^61,95^ in which the torque generated by gravity is compensated by hip abductor activity to regulate angular momentum and ensure frontal upper-body balance^96–98^. Since the AMP also generated roll torque components, it is likely that also frontal balance control strategies were used. This would probably result in altered hip muscle activities (depending on the corresponding perturbation torque) as found by previous multidirectional perturbation studies when applying surface transitions^38,99–102^ and rotations^102,103^. Given that each leg individually controls the CoP^61^, cross-talk from the contralateral leg to ipsilateral ankle joint muscles should be small. For the hip, however, neuronal coupling of both legs – next to their mechanical interaction – e.g. by interneurons is very likely. Still, muscular responses did not appear to differ (see Supplementary Figs S12–S14) allowing us to merge both conditions of inverted roll but similar pitch perturbations.

To generate a rotation of the gimbal wrt. to the body frame of the subjects, a peak gimbal torque of 12 Nm was necessary. This torque created an angular acceleration (Eq. (2)) that resulted in upper-body yaw rotations of 3° to 5° (see Supplementary Fig. S8). This could especially influence GLU and TFL activity, as both muscles also function to generate a leg rotation in the transversal plane (wrt. to the pelvis). However, since the observed upper-body yaw was small compared to our main perturbation direction, such influence is expected to be small. A future version of the AMP may use instead two or more smaller actuators to allow both the magnitude and direction of the torque vector to be controlled simultaneously, thereby eliminating torques in unintended directions^104^ and allowing multi-directional perturbation studies^38,83^.

A drawback of the selected actuation principle is that the mass of the actuator is borne by the test subject and cannot be placed externally. The prototype AMP in this study weighed 16 kg, or 15% to 29% of the participant’s body weight. Other studies of standing with similar backpack load showed tendencies of increasing activity for anterior and reductions for posterior muscles groups. In particular, non-significant increases of VL, RF and reduced HAM activity as well as increased CoP sway were found^105,106^. In our study, significant reductions in muscle activity were only seen in GLU, HAM of the left leg and VL of the right leg. Still, we cannot exclude other influences of e.g. modulation of muscular stiffness or related effects on balance. Also, an increased hip flexion (by 6.2°), more extended knee (by 2.1°) and increased CoP sway were found (see Supplementary Table S1 and Fig. S7). The additional weight makes the balance control more challenging^106^. When being perturbed, it is likely that the added moment of inertia of the AMP helped subjects to maintain the initial upper-body posture. The influence of muscle fatigue should be small, since subjects had sufficient time to rest. Additionally, also the generated vibrations or the noise could result in changed muscle activity, e.g. by stimulating muscle-spindle proprioception^107,108^ or psychological factors like attention or anxiety^109–112^.

In future generations of the AMP it will be possible to reduce the mass substantially by (i) using lightweight components, (ii) scaling down the maximum possible perturbation magnitude, (iii) increasing the diameter of the flywheel, or (iv) rotating the flywheel faster. Lightweight reactionless actuators that are wearable^90,91,94,113^ or handheld^114,115^ have been recently developed, and the technology has even been demonstrated on the sub-gram scale^116^. However, for the same perturbation magnitude, we estimate that it is currently feasible to construct a AMP weighing approximately half the one used in this study.

## Conclusion

This study investigated the specific role of biarticular muscles in dealing with upper-body pitch perturbations in human standing. Our main finding was that biarticular thigh muscles (hamstring and rectus femoris) showed the strongest response of all measured major leg muscles. Our results indicate a reactive control for biarticular thigh muscles in line with a previously suggested torque control strategy considering the difference between hip and knee torques. To the knowledge of the authors, this was the first study on human subjects providing experimental evidence that biarticular muscles play a key role in reactive upper-body balance control.

In order to focus on upper-body balance while reducing method-dependent artefacts (arising e.g. from inter-segmental couplings or global perturbations), a new type of perturbation was applied by the Angular Momentum Perturbator (AMP). Complementing existing perturbation devices, the generated torque perturbation at the trunk provides a new methodology for studying human response strategies.

Future research will involve applying mechanical and neuromechanical models^20,46,51,117,118^ and/or data-driven approaches^119^ to increase our understanding of underlying control mechanisms as well as classification of user intention, e.g. for controlling wearable robotic devices like the AMP.

A deeper understanding of postural balance control of the upper body and involved muscle responses will facilitate the design of robotic systems for assistance or rehabilitation and improve the effectiveness of physical training for fall prevention e.g. for elderly people.

## Supplementary information

Supplementary Material

## Data Availability

Processed datasets of the AMP, kinematic, kinetic, electromyographic and anthropometric data that were used in this study are available in the 4TU.ResearchData repository^120^.
