# Supplementary material for: Biarticular muscles are most responsive to upper-body pitch perturbations in human standing
Source: Sci Rep. 2019 Oct 10;9:14492. doi: 10.1038/s41598-019-50995-3 (PMC6787002; doi:10.1038/s41598-019-50995-3)
Supplement: Supplementary file 1 — Supplementary Material [file 41598_2019_50995_MOESM1_ESM.pdf]

Biarticular muscles are most responsive to upper-body pitch perturbations in human standing – Supplementary Material

Christian Schumacher[1,2,*], Andrew Berry[2], Daniel Lemus[2], Christian Rode[3], André Seyfarth[1], and Heike Vallery[2]

[1]Lauflabor Locomotion Laboratory, Institute of Sport Science, Centre for Cognitive Science, Technische Universität Darmstadt, Darmstadt, 64289, Germany
[2]Delft Biorobotics Lab, BioMechanical Engineering, Delft University of Technology, Delft, 2628 CD, The Netherlands
[3]Friedrich-Schiller-Universität Jena, Institute of Zoology and Evolutionary Research, Jena, 07743, Germany
[*]schumacher@sport.tu-darmstadt.de

Experimental setup

In August and September 2018, we collected kinematic, kinetic and electromyographic (EMG) data in the Delft Biorobotics Lab. All measurement devices including the data logging of the AMP were synchronized by a 5 V trigger signal.

The used marker and EMG electrode locations can be found in Fig. 1.

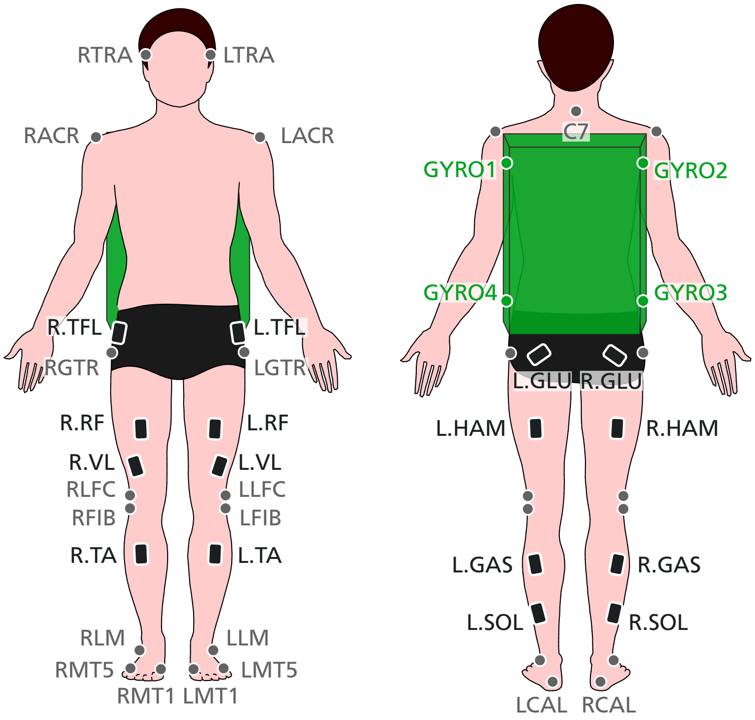

Figure 1. Location of EMG sensors and used marker model. EMG electrodes (black) and passive motion capture markers: body markers (grey) and AMP (green).

Mechanical analysis of all subjects

To evaluate the body posture after perturbations were applied, joint angles are shown in Fig. 2. The resultant body postures of positive and negative torque perturbations showed slightly different response behavior. The subjects' mechanical response consisted of a distinct early trunk pitch (hip flexion, within first 500 ms) while other major leg joints, knee and ankle, were only slightly flexed or extended, respectively. In the case of negative perturbations, subjects mainly bend their knees resulting in backwards motion of the trunk and thigh instead of a clearly extended hip (only 5° to 10°).

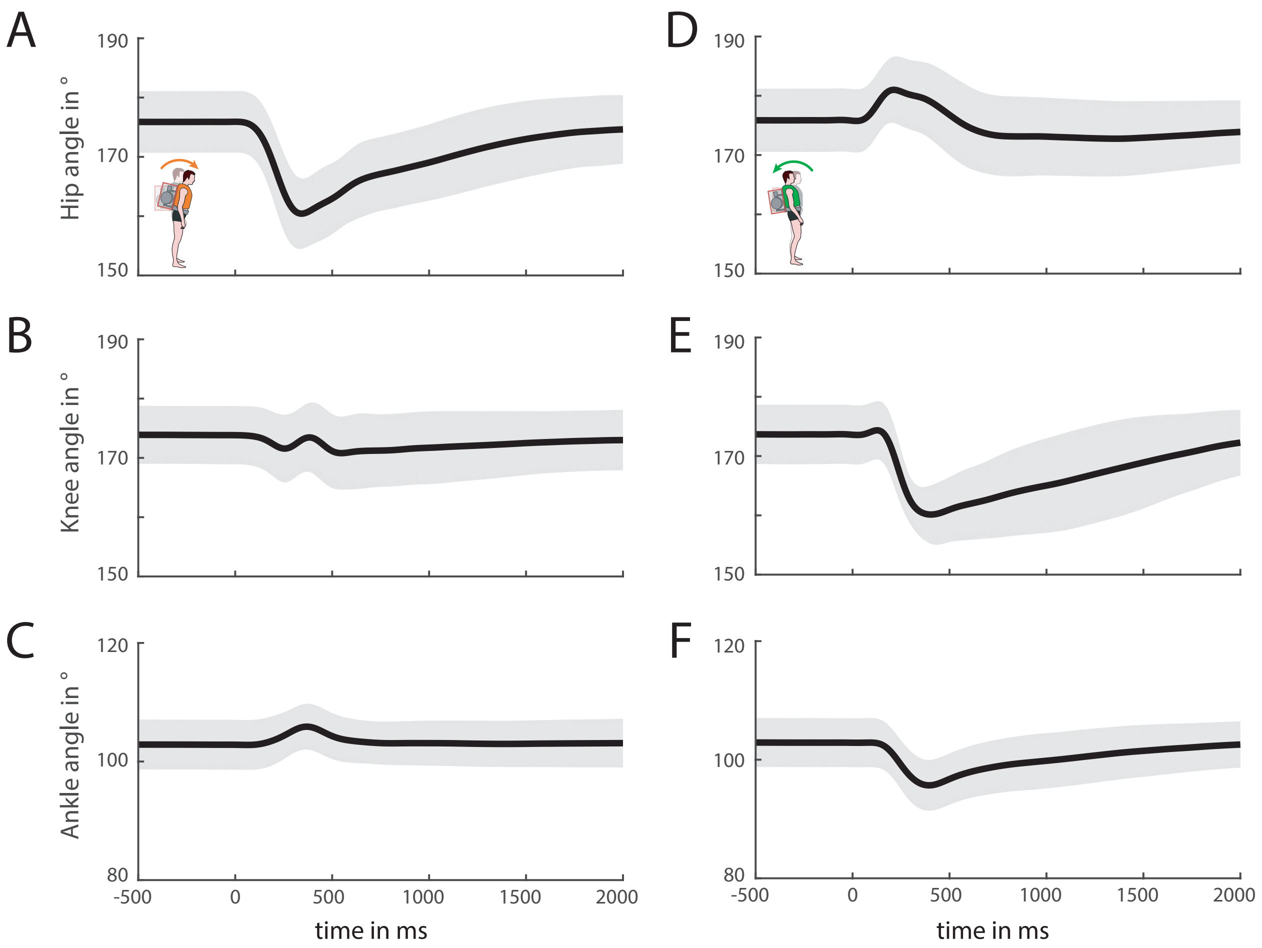

Figure 2. **Grand mean and SD of major sagittal joint angles of all eleven subjects.** Hip **(A, D)**, knee **(B, E)** and ankle angles **(D, F)** of positive (left side) and negative torques (right side) are shown. Increasing joint angle changes denote a joint extension.

The following figures show the resultant motion of all subjects. Fig. 3, 4 5 and 6 show the mean changes in trunk lean, hip joint position CoM position and CoP position of all subjects in ap-direction, respectively. Observed response patterns were quite similar with some intra-subject variability.

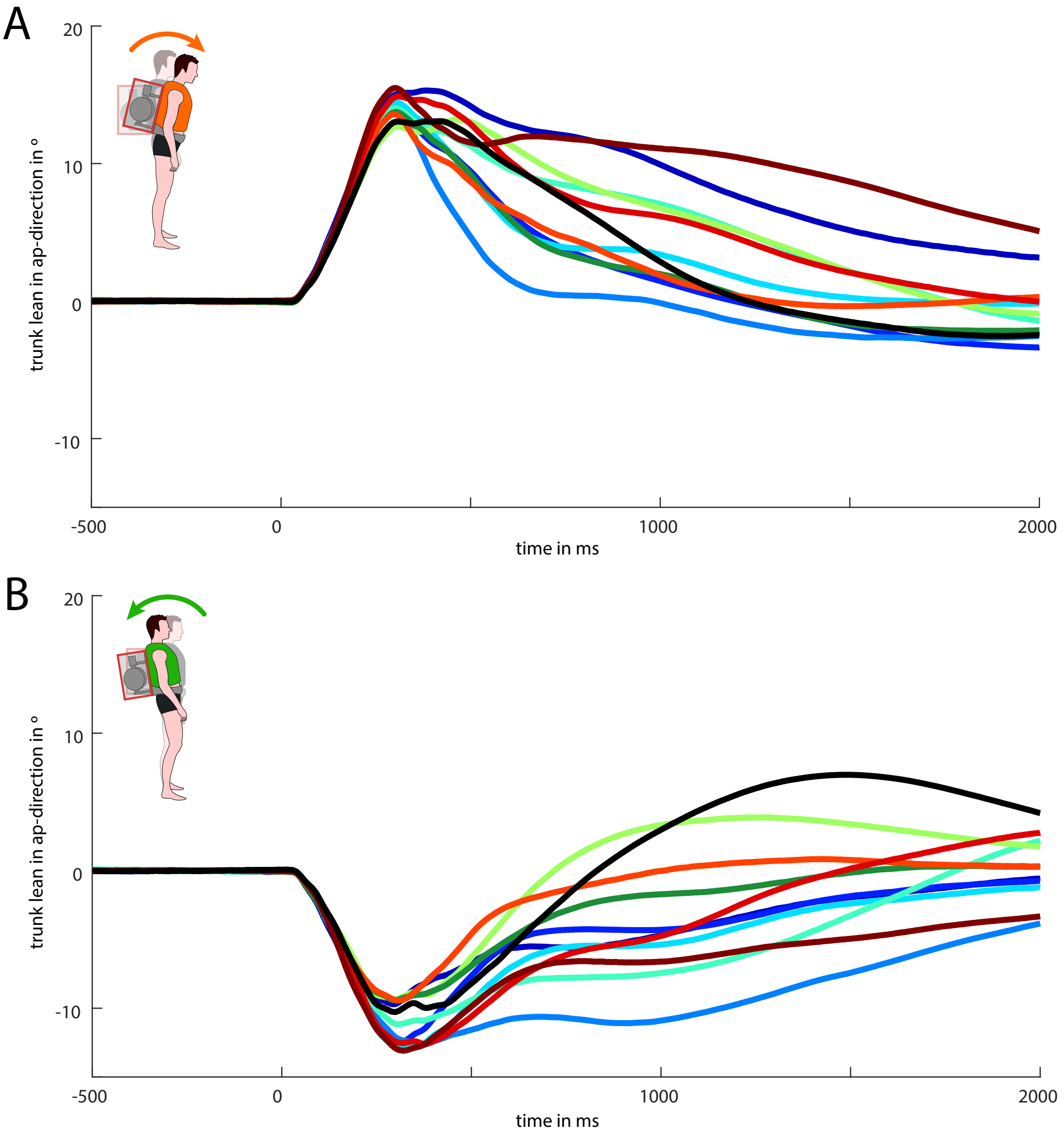

Figure 3. Change in mean trunk lean in ap-direction of all eleven subjects in positive (A) and negative (B) direction. The single subject shown in the manuscript is colored black here.

The applied torques are equal to a force pair with equal magnitudes and opposing directions. In forward perturbations, the shoulder and hip joints were accelerated forwards and backwards, respectively. Fig. 4 shows the resultant hip joint position of all subjects. In positive perturbations, the hip joint moved posterior by about 5 cm, while, in negative perturbations, slightly less anterior movement was observed.

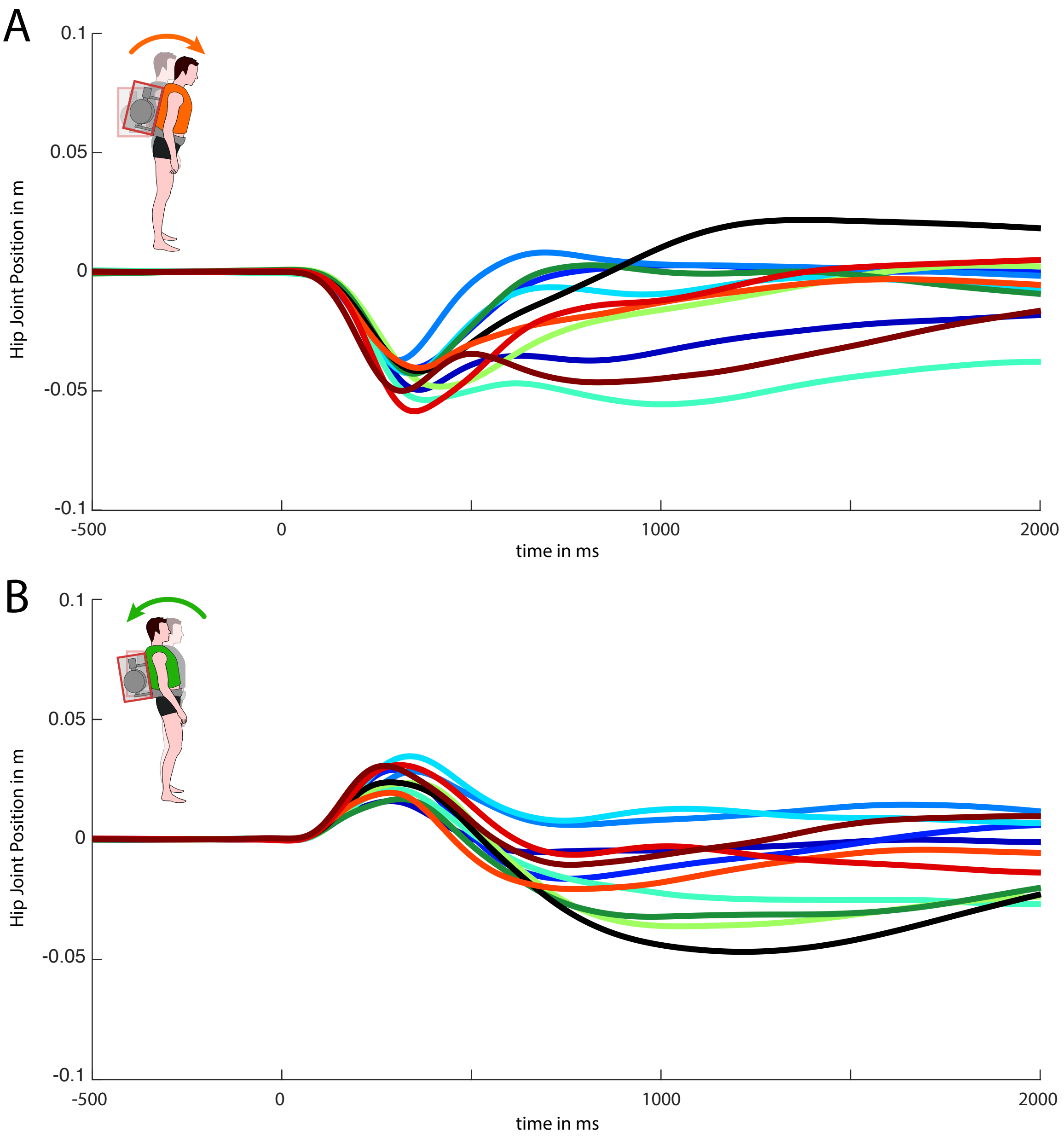

Figure 4. Change in mean hip joint position in ap-direction of all eleven subjects in positive (A) and negative (B) direction. The single subject shown in the manuscript is colored black here.

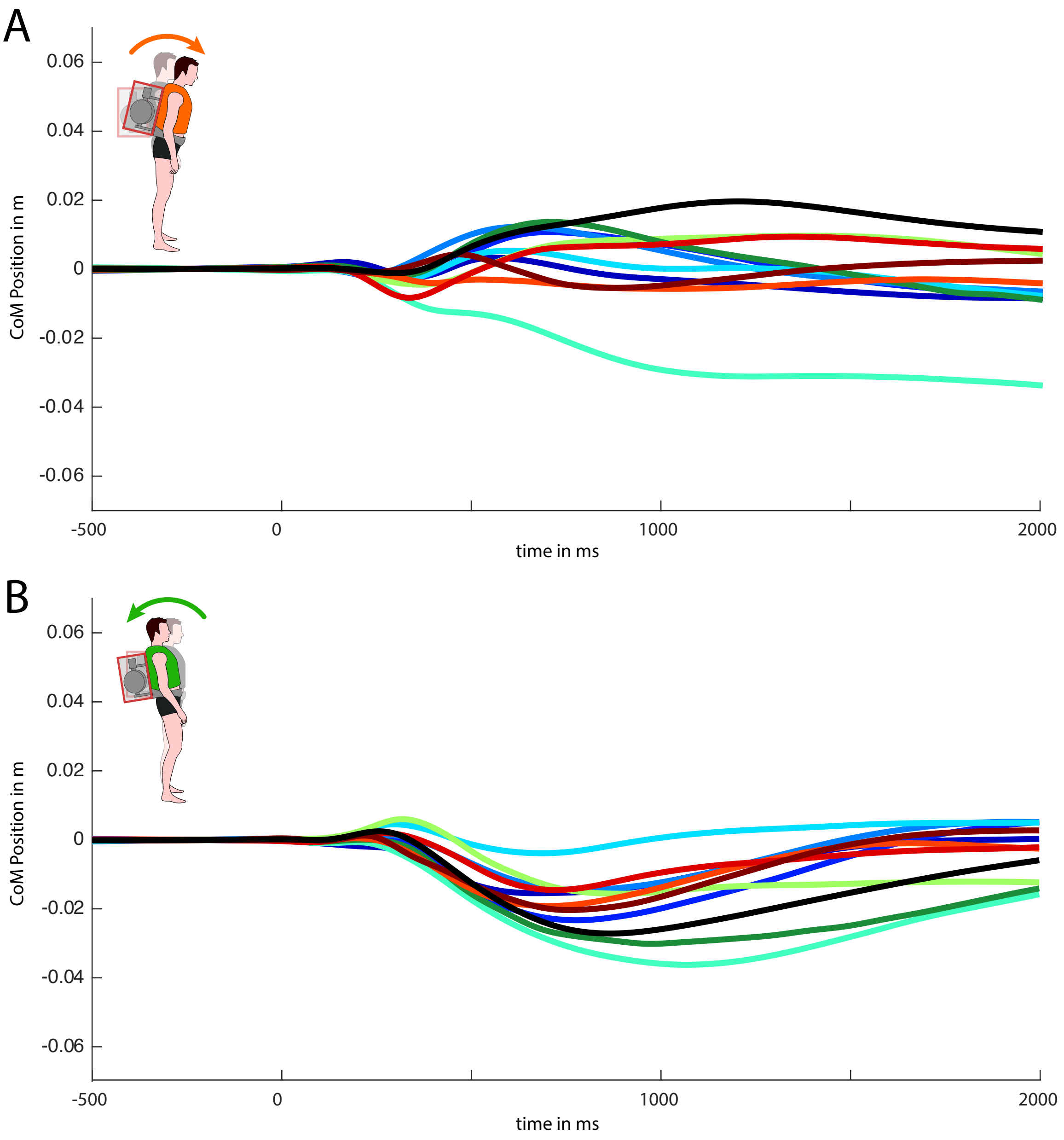

Figure 5. Change in mean CoM position in ap-direction of all eleven subjects in positive (A) and negative (B) direction. The single subject shown in the manuscript is colored black here.

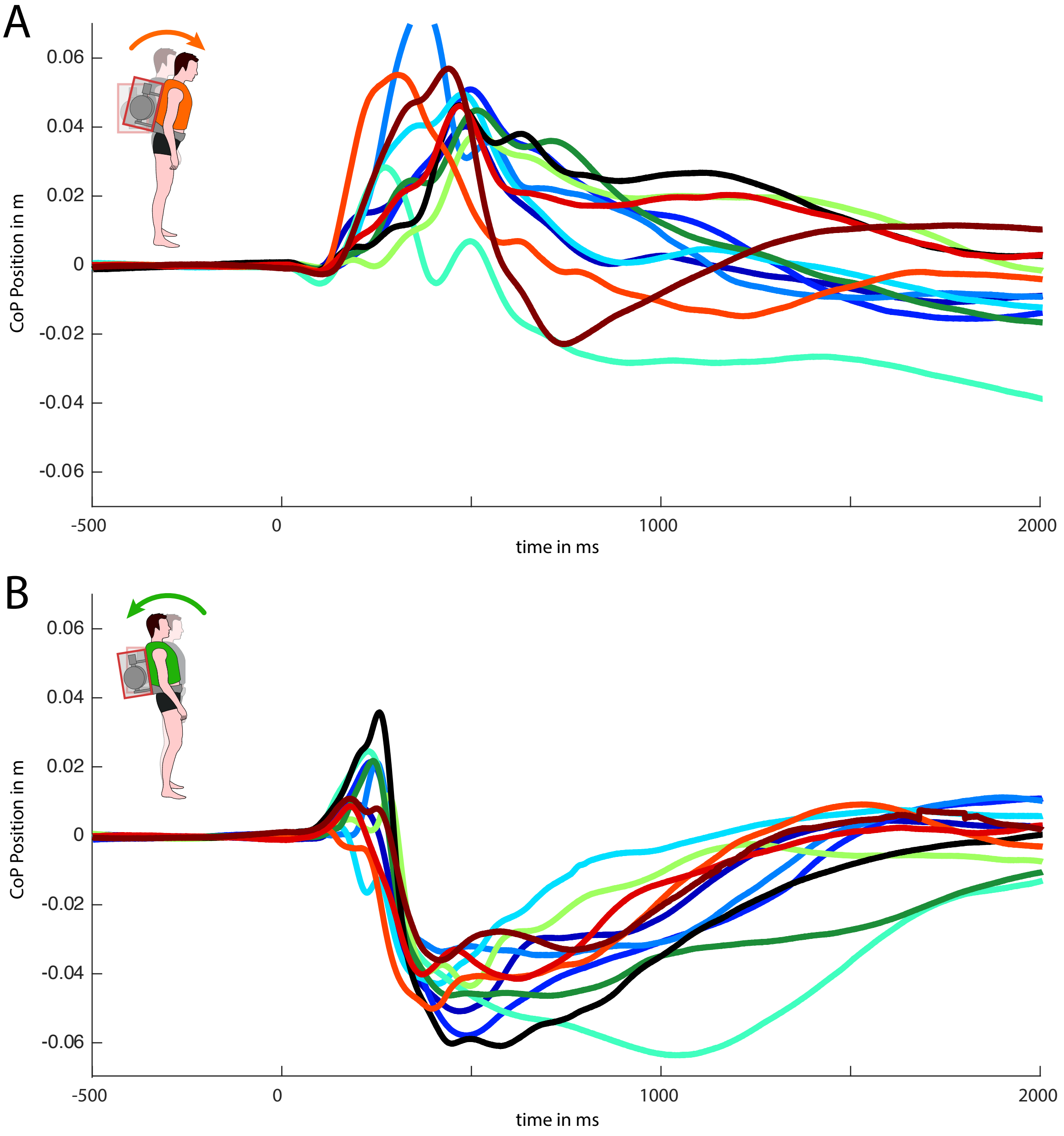

Figure 6. Change in mean CoP position in ap-direction of all eleven subjects in positive (A) and negative (B) direction. The single subject shown in the manuscript is colored black here.

Influence of AMP weight, vibration and noise

To check to what extent the weight, vibration and noise of the AMP may have influenced the initial posture, mean joint angles in the 'Unloaded Standing' and 'Loaded Standing' conditions of all major joints are shown in Table 1. Subsequently, wearing the AMP resulted in a slightly more bend hip ($-6.2°$) and more extended knee ($2.1°$).

Table 1. Comparison of postural changes due to AMP weight, vibration and noise. Mean major joint angles (in °) of all subjects of 20 s periods of quiet standing in the 'Unloaded Standing' and 'Loaded Standing' conditions. $\Delta\phi$ denotes mean change. For normally distributed comparisons, the paired two-sided t-test was used. In case of non-normal comparisons, the non-parametric two-sided Wilcoxon signed-rank test was used. Significant values ($p < 0.05$) are highlighted by bold text.

Joints	'Unloaded Standing'	'Loaded Standing'	$\Delta\phi$	p-value
Hip	183.6	177.4	-6.2	**2.52E-04**
Knee	172.3	174.4	+2.1	**0.002**
Ankle	102.4	102.9	+0.4	0.235

We further compared the CoP trajectory of both conditions of 'Unloaded Standing' and 'Loaded Standing' (Fig. 7). Here, for most subjects a slight increase in CoP sway can be observed, agreeing with results from[1,2].

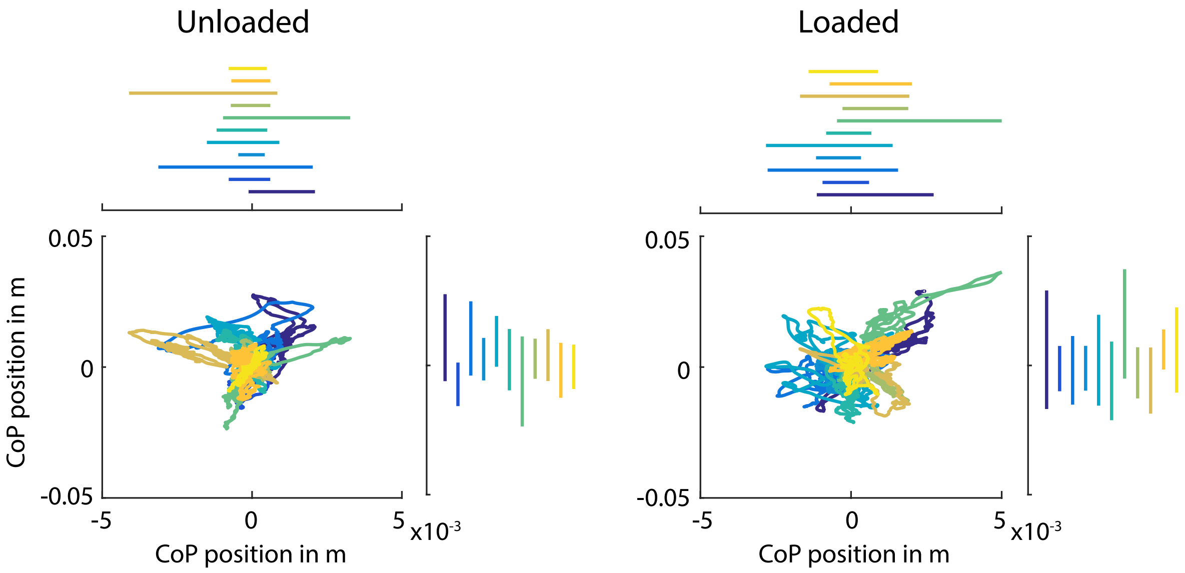

Figure 7. Change in CoP position of all eleven subjects during 20 s intervals of 'Unloaded Standing' and 'Loaded Standing' measurements. Bars denote the maximum range of CoP sway. For each subject a different color is used.

Influence of Gimbal torque

To further check the influence of the gimbal torque, the trunk yaw rotation (around the longitudinal axis of the trunk) is shown in Fig. 8. Here, for different conditions of initial gimbal position, the change in trunk yaw resulted in trajectories of opposing direction with a magnitude of 3° to 5°.

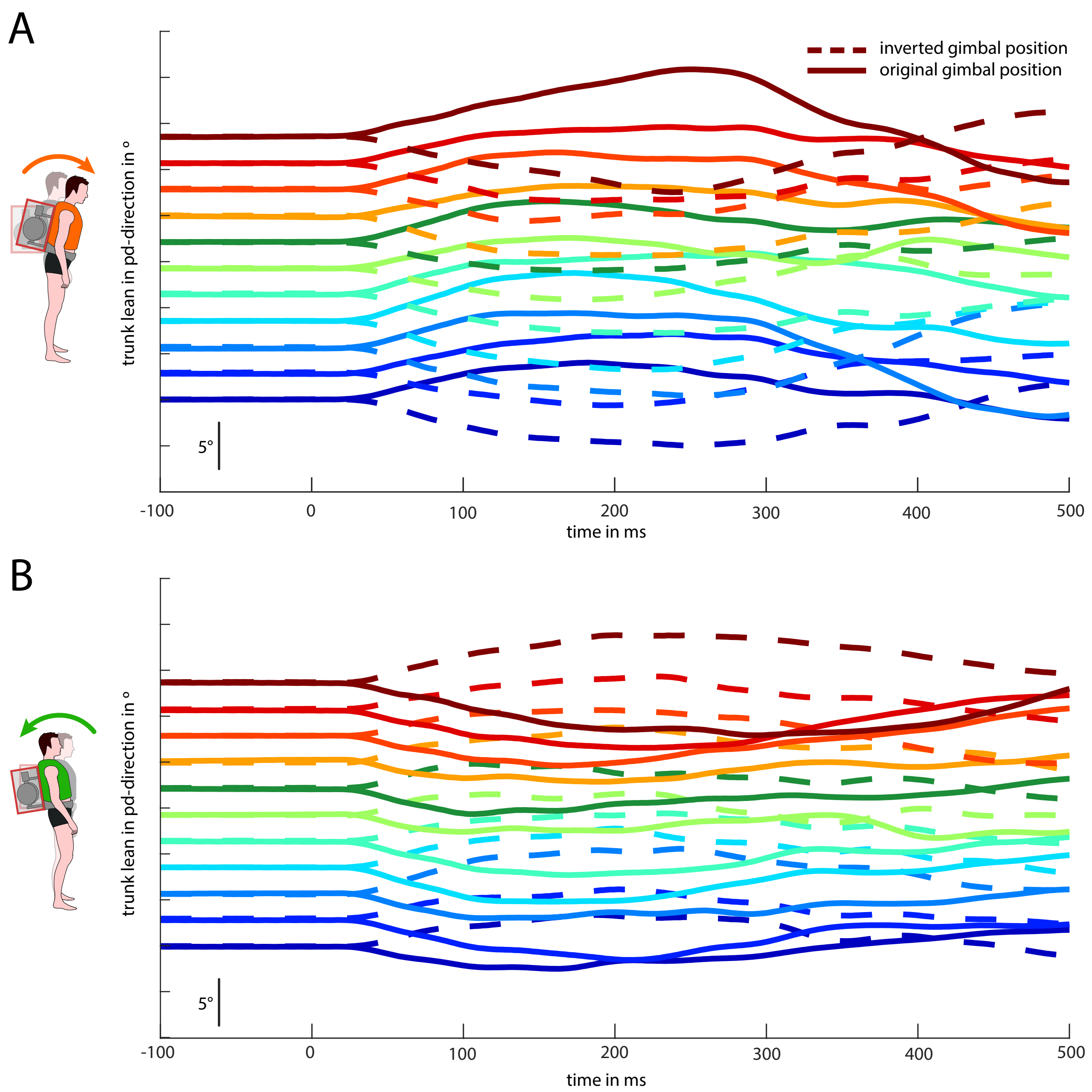

Figure 8. **Change of mean trunk yaw of all eleven subjects in different conditions of initial gimbal positions:** positive **(A)** and negative perturbation **(B)**. Solid and dashed lines denote the original and inverted gimbal position, respectively.

Influence of roll torque component

In Fig. 9 the resultant trunk roll about the frontal plane for all four conditions is shown (positive and negative torque perturbation, original and inverted gimbal position). For positive perturbation directions, a maximum of 8° of trunk roll was found while most subjects leaned by only 2° to 4°. Up to 4° of trunk roll can be observed for the negative perturbation. Both conditions of original and inverted gimbal positions show trunk rolls with similar magnitudes but opposing directions. For positive torques, higher magnitudes and delayed timings of peak deflections occurred compared to negative torque perturbations.

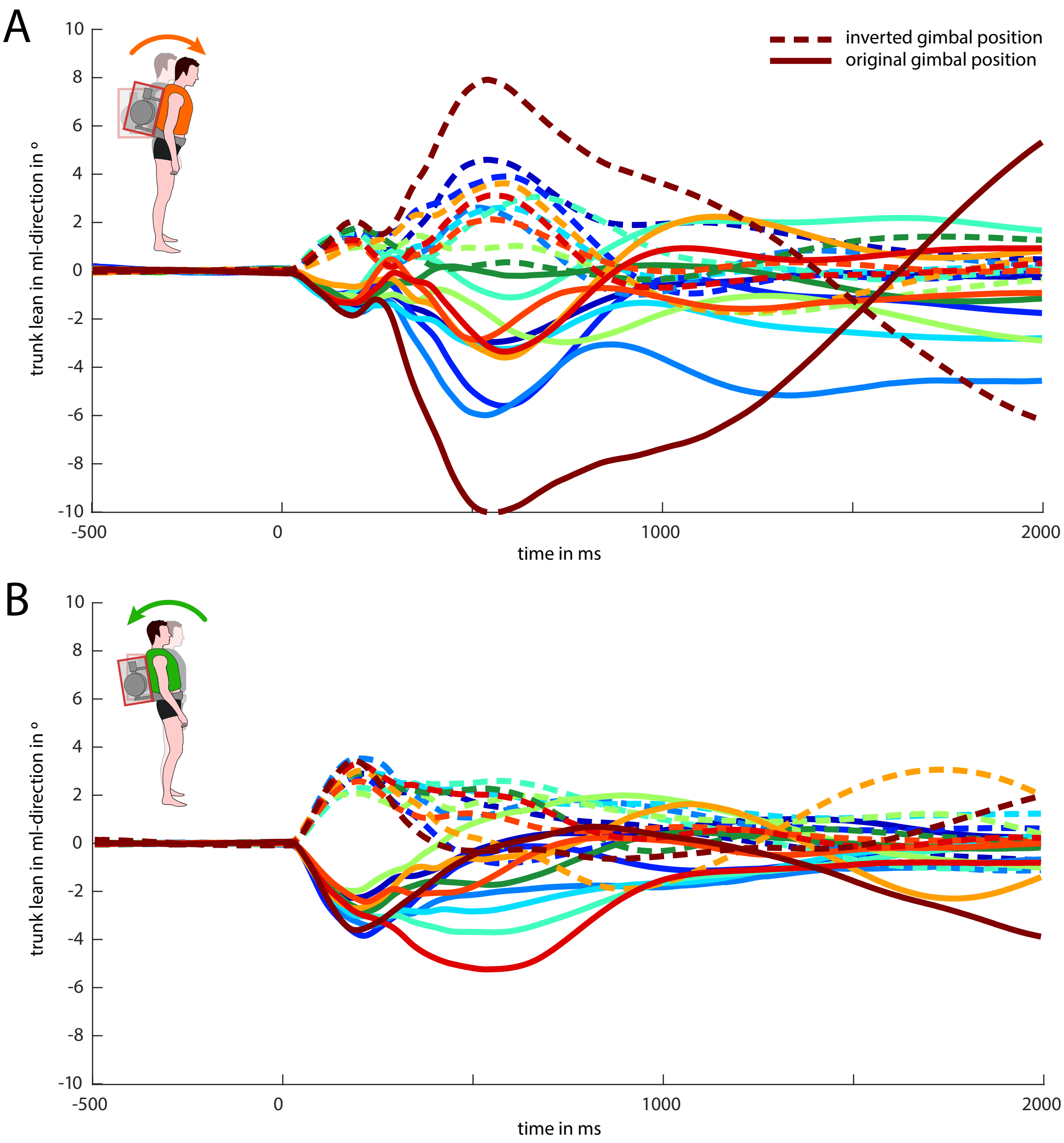

Figure 9. Change of mean trunk roll of all eleven subjects in different conditions of initial gimbal positions: positive **(A)** and negative perturbation **(B)**. Solid and dashed lines denote the original and inverted gimbal position, respectively.

Also the corresponding leg loading as measured by the vertical GRF show changes in opposite directions but with similar magnitudes for both conditions of inverted roll torque components, starting after about 50 ms to 100 ms (Fig. 10).

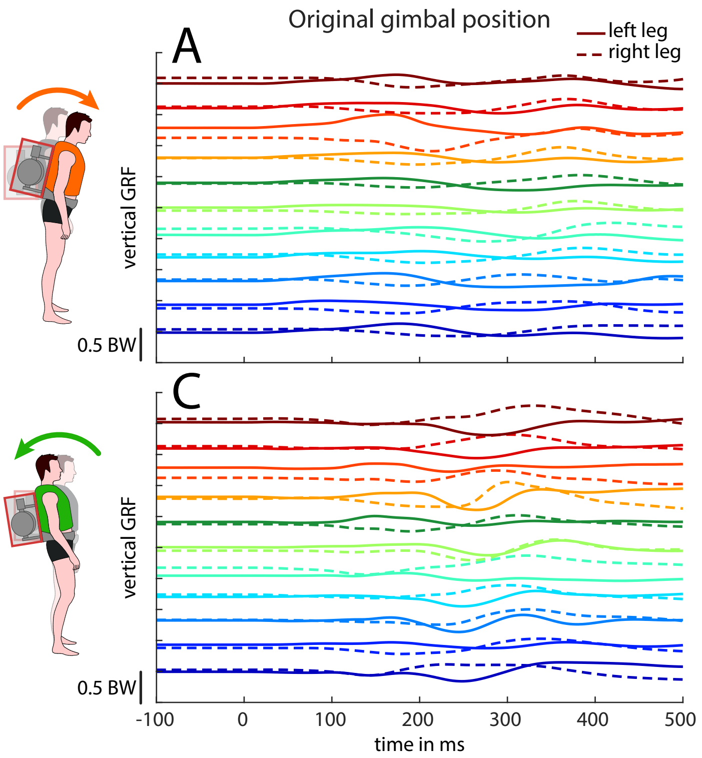

Figure 10. Change in mean leg loading (vertical GRF) of all eleven subjects in different conditions: positive with original gimbal position **(A)**, positive with inverted gimbal position **(B)**, negative with original gimbal position **(C)**, negative with inverted gimbal position **(D)**. Solid and dashed lines denote the left and right leg, respectively.

In positive perturbation direction, this change in leg loading had only slight impact on the CoP positions (Fig. 11A). Both legs still resemble quite similar trajectories. Bigger differences between both legs can be seen in the negative perturbations (Fig. 11B).

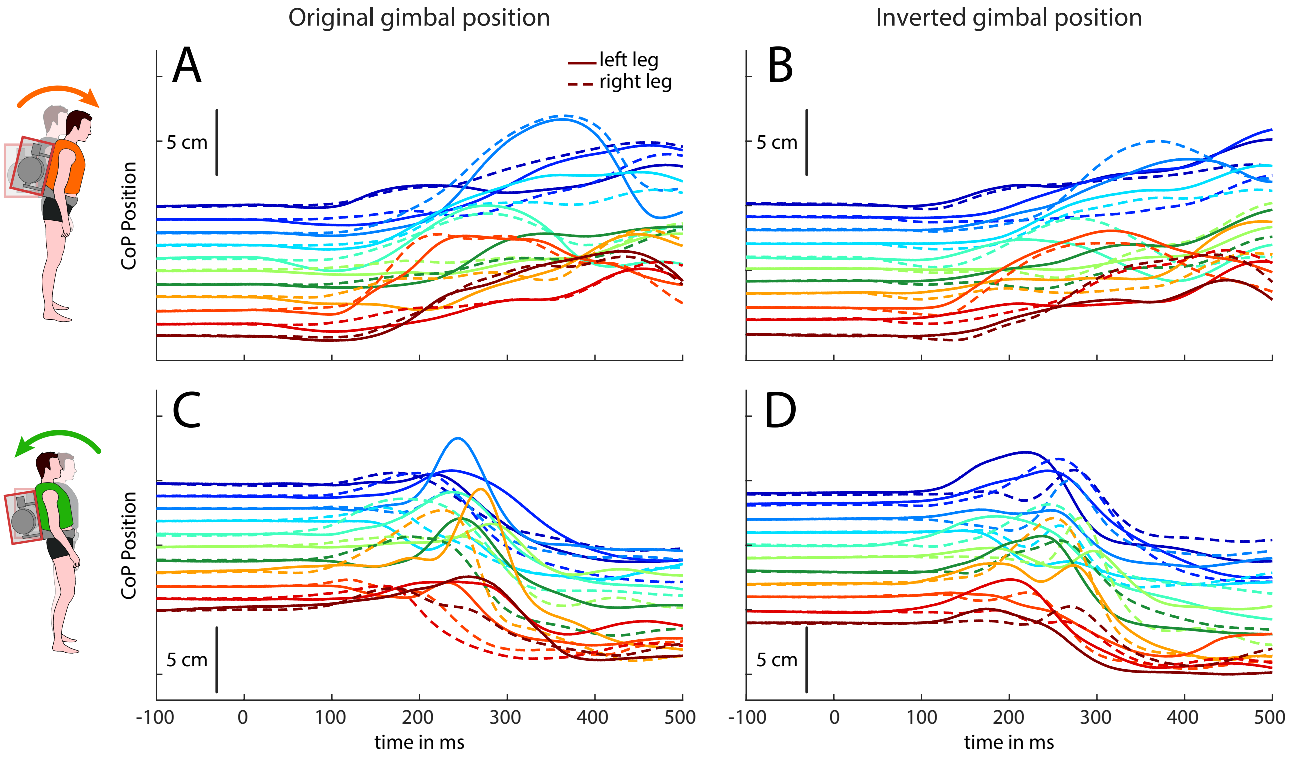

Figure 11. Change in mean CoP position in ap-direction of all eleven subjects in different conditions: positive with original gimbal position **(A)**, positive with inverted gimbal position **(B)**, negative with original gimbal position **(C)**, negative with inverted gimbal position **(D)**. Solid and dashed lines denote the left and right leg, respectively.

To further check influences of the roll torque component on the muscular response, muscular activity levels of both legs of all conditions are compared. Fig. 12 to Fig. 14 denote the responses in the corresponding response intervals RI1, RI2 and RI3.

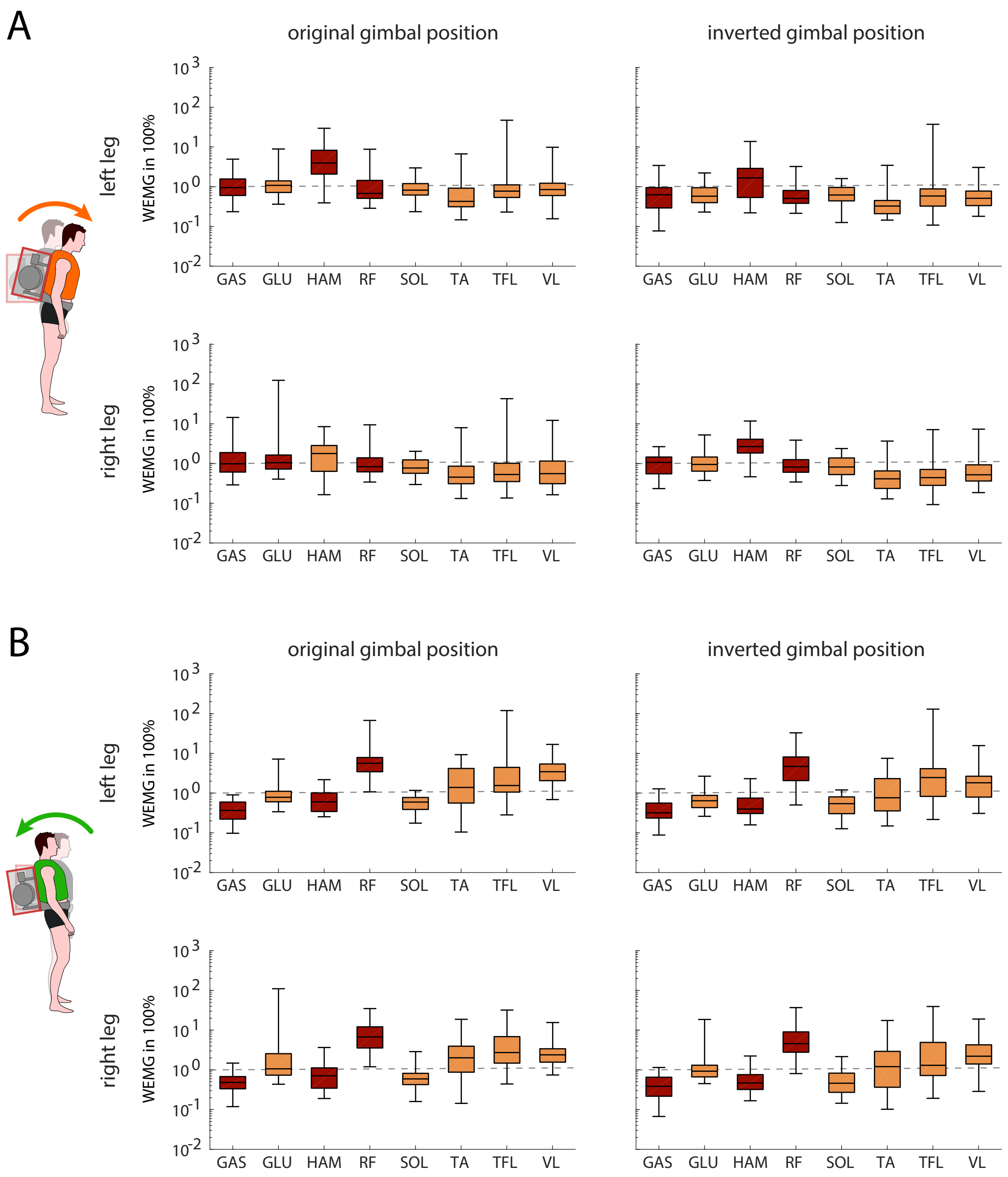

Figure 12. Comparison of muscular activity levels in response interval RI1 (100 − 150 ms) **of all conditions and both legs:** Boxplots (pooled data of all analyzed trials) of the normalized and mean response amplitudes (WEMG) for positive torque perturbations **(A)** and negative torque perturbations **(B)**, left (upper panels) and right leg (bottom panels) and original (left side) and inverted gimbal position (right side). Results of mono- (yellow) and biarticular muscles (red) are presented in a logarithmic scaling. Mean EMGs were normalized by the individuals mean muscle stimulation during walking trials (see Methods).

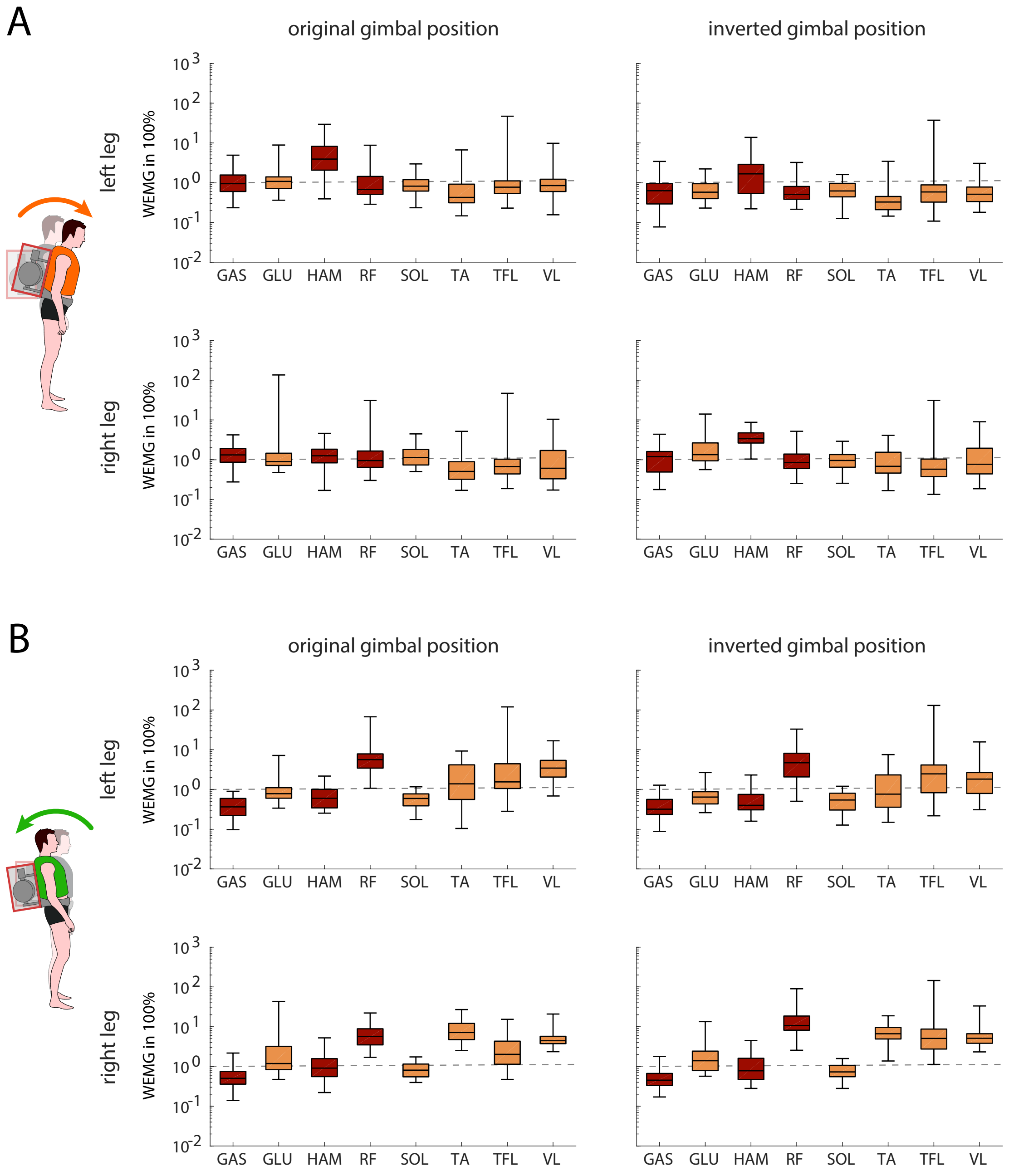

Figure 13. Comparison of muscular activity levels in response interval RI2 (170 − 250 ms) **of all conditions and both legs:** Boxplots (pooled data of all analyzed trials) of the normalized and mean response amplitudes (WEMG) for positive torque perturbations **(A)** and negative torque perturbations **(B)**, left (upper panels) and right leg (bottom panels) and original (left side) and inverted gimbal position (right side). Results of mono- (yellow) and biarticular muscles (red) are presented in a logarithmic scaling. Mean EMGs were normalized by the individuals mean muscle stimulation during walking trials (see Methods).

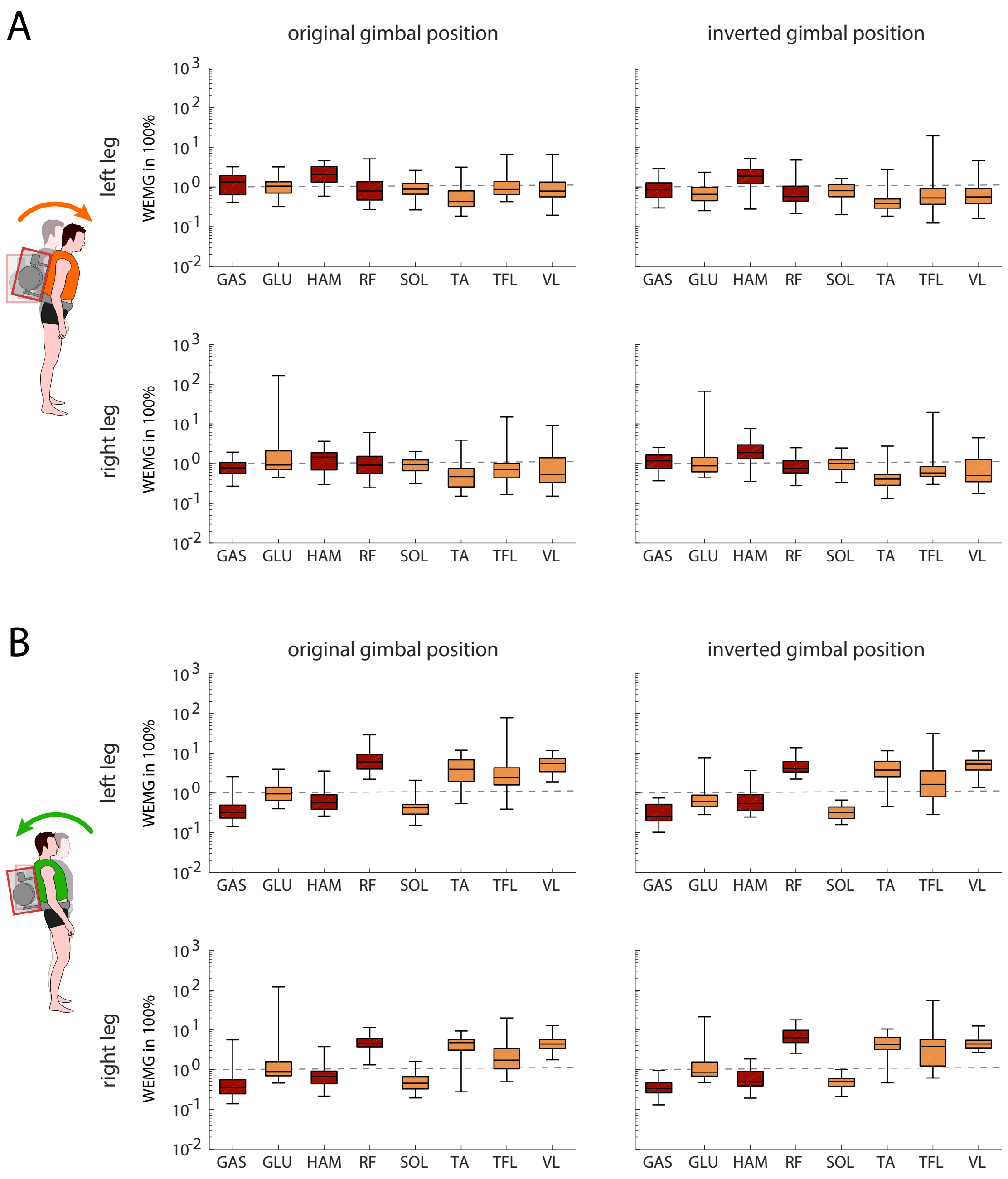

Figure 14. Comparison of muscular activity levels in response interval RI3 (270 − 350 ms) **of all conditions and both legs:** Boxplots (pooled data of all analyzed trials) of the normalized and mean response amplitudes (WEMG) for positive torque perturbations **(A)** and negative torque perturbations **(B)**, left (upper panels) and right leg (bottom panels) and original (left side) and inverted gimbal position (right side). Results of mono- (yellow) and biarticular muscles (red) are presented in a logarithmic scaling. Mean EMGs were normalized by the individuals mean muscle stimulation during walking trials (see Methods).

Based on these results (Fig. 12 to Fig. 14), only slight differences of muscular response amplitudes were observed for both conditions of initial gimbal position.

Comparison of muscular prestimulation of left and right leg

Table 2 shows the calculated changes and significance levels from comparisons of ‘loaded Standing’ and mean ‘Pre-Perturbation’ activations of both legs.

Table 2. Comparison of left and right leg prestimulation. Mean and SD changes (Δ in WEMG) and p-values of normalized and mean response amplitudes for comparisons of ‘Unloaded Standing’ (UL), ‘Loaded Standing’ (L) and the mean ‘Pre-Perturbation’ activation for all 10 trials (averaged over last 5 trials per condition $\times$ 2 gimbal configurations) for all 11 subjects. For comparisons, the paired two-sided t-test (for normal distribution) or the non-parametric two-sided Wilcoxon signed-rank test (if assumption of normality was violated) was used. Significant values ($p < 0.05$) are highlighted by bold text.

Side	Muscle	‘L’ vs. ‘UL’		‘PP’ vs. ‘L’	
		Δ WEMG	p-values	Δ WEMG	p-values
left	GLU	-20 ±22	**0.003**	+8 ±13	**0.033**
	HAM	-46 ±57	**0.013**	-5 ±14	0.308
	GAS	+2 ±03	0.061	0 ±11	0.213
	SOL	+7 ±12	0.078	-2 ±07	0.345
	TFL	+7 ±12	0.155	+5 ±07	0.065
	RF	+10 ±36	0.790	-9 ±48	0.790
	VL	+1 ±07	0.730	0 ±28	0.213
	TA	0 ±03	0.695	-1 ±08	0.182
right	GLU	-7 ±35	0.051	-20 ±41	0.091
	HAM	-7 ±42	0.091	-5 ±30	0.930
	GAS	+1 ±16	0.862	+1 ±10	0.792
	SOL	+8 ±13	0.058	+2 ±07	0.354
	TFL	+19 ±30	0.061	-15 ±22	**0.048**
	RF	+8 ±31	0.790	-30 ±44	0.051
	VL	-10 ±13	**0.027**	-8 ±14	0.075
	TA	+1 ±03	0.364	-2 ±04	0.098

Comparison of muscular reflex response of positive and negative perturbations

Table 3 shows the comparisons (mean differences and significance levels) of the reflex responses of all muscles. For positive perturbation, HAM responded with significantly higher activity levels than any other muscle. In negative perturbations, anterior and posterior muscle groups were significantly different. Additionally, RF stimulation was higher than all other muscles.

Table 3. Comparison of relative reflex responses for positive (upper panel) and negative (lower panel) perturbations. Results from the rmANOVA post-hoc test of the interaction effect 'Muscle' x 'Direction' ($F(7,314) = 65.2, p < 0.001$). Mean differences (upper value, in WEMG) and p-values (lower value) of comparisons between muscles per perturbation direction (over both legs and all response intervals). Comparisons were done for the averaged relative reflex responses of the left leg (last 5 trials per condition × 2 gimbal configurations) for all 11 subjects. Significant values ($p < 0.05$) are highlighted by bold text.

positive perturbation	HAM	GAS	SOL	GLU	TFL	TA	VL	RF
HAM	-	+117	+156	+190	+177	+184	+166	+193
	-	**< 0.001**	**< 0.001**	**< 0.001**	**< 0.001**	**< 0.001**	**< 0.001**	**< 0.001**
GAS		-	+39	+72	+60	+67	+49	+76
		-	0.942	0.378	0.637	0.483	0.833	0.318
SOL			-	+34	+21	+28	+10	+37
			-	0.974	0.999	0.990	1.000	0.956
GLU				-	−13	−5	−24	+3
				-	0.999	1.000	0.997	1.000
TFL					-	+7	−11	+16
					-	1.000	1.000	0.999
TA						-	−18	+9
						-	0.999	1.000
VL							-	+27
							-	0.993
RF								-
								-

negative perturbation	HAM	GAS	SOL	GLU	TFL	TA	VL	RF
HAM	-							
	-							
GAS	−17	-						
	0.999	-						
SOL	−11	+6	-					
	1.000	1.000	-					
GLU	+1	+18	+12	-				
	1.000	0.999	1.000	-				
TFL	+257	+274	+268	+256	-			
	**< 0.001**	**< 0.001**	**< 0.001**	**< 0.001**	-			
TA	+325	+342	+337	+324	+69	-		
	**< 0.001**	**< 0.001**	**< 0.001**	**< 0.001**	0.452	-		
VL	+385	+402	+396	+383	+128	+59	-	
	**< 0.001**	**< 0.001**	**< 0.001**	**< 0.001**	**0.004**	0.628	-	
RF	+586	+603	+597	+585	+329	+261	+202	-
	**< 0.001**	**< 0.001**	**< 0.001**	**< 0.001**	**< 0.001**	**< 0.001**	**< 0.001**	-

Peak to mean ratios of background walking activity

Table 4 provides ratios of the filtered background activity during unloaded level walking to convert the mean background activity (WEMG, this study) to peak background activity (for comparisons with other studies).

Table 4. EMG Ratio of peak to mean background walking activity of all muscles averaged over 10 trials.

Muscle	left Side	right Side
GLU	6.72	6.65
HAM	7.30	8.04
GAS	5.72	8.87
SOL	4.29	6.90
TFL	5.00	6.37
RF	5.32	5.23
VL	7.85	10.99
TA	7.29	6.14

References

1. Al-Khabbaz, Y. S., Shimada, T. & Hasegawa, M. The effect of backpack heaviness on trunk-lower extremity muscle activities and trunk posture. *Gait & posture* **28**, 297–302 (2008).

2. Schiffman, J. M., Bensel, C. K., Hasselquist, L., Gregorczyk, K. N. & Piscitelle, L. Effects of carried weight on random motion and traditional measures of postural sway. *Appl. Ergonomics* **37**, 607–614 (2006).
